# Empagliflozin improves post-infarction cardiac remodeling through GTP enzyme cyclohydrolase 1 and irrespective of diabetes status

**DOI:** 10.1038/s41598-020-70454-8

**Published:** 2020-08-11

**Authors:** Maria del Carmen Asensio Lopez, Antonio Lax, Alvaro Hernandez Vicente, Elena Saura Guillen, Antonio Hernandez-Martinez, Maria Josefa Fernandez del Palacio, Andoni Bayes Genis, Domingo A. Pascual Figal

**Affiliations:** 1 Biomedical Research Institute Virgen de La Arrixaca (IMIB-Arrixaca), University of Murcia, Ctra. Madrid-Cartagena S/N, 30120 Murcia, Spain; 2 Cardiology Department, IMIB-Arrixaca, University of Murcia, Hospital Virgen de la Arrixaca, Murcia, Spain; 3 Endocrinology Department, Hospital Virgen de La Arrixaca, University of Murcia, Murcia, Spain; 4 Veterinary Medicine and Surgery Department, Veterinary Teaching Hospital, University of Murcia, Murcia, Spain; 5 Heart Institute, Hospital Universitari German Trías i Puyol, CIBERCV, BadalonaMadrid, Spain; 6 Cardiology Department, IMIB-Arrixaca, University of Murcia, Hospital Virgen de la ArrixacaLAIB room 2.52, Avda. Buenavista s/n, 30120 Murcia, Spain; 7 Centro Nacional de Investigaciones Cardiovasculares (CNIC), Madrid, Spain; 8 CIBERCV, Madrid, Spain

**Keywords:** Cardiovascular diseases, Translational research, Cardiology, Medical research

## Abstract

Sodium-glucose co-transporter-2 inhibitors (SGLT2i) have shown to prevent heart failure progression, although the mechanisms remain poorly understood. Here we evaluated the effect of empagliflozin (EMPA, SGLT2i) in cardiac remodeling after myocardial infarction, the interplay with diabetes status and the role of cardiac GTP enzyme cyclohydrolase 1 (cGCH1). A rat model of diabetes (50 mg/kg streptozotocin, i.p.) was subjected to myocardial infarction and left ventricular systolic dysfunction, by ligation of the left anterior descending coronary artery. EMPA therapy significantly improved cardiac remodeling parameters and ameliorated processes of fibrosis and hypertrophy, in both non-diabetic and diabetic rats. This cardioprotective effect related with a significant increase in myocardial expression levels of cGCH1, which led to activation of nNOS and eNOS, and inhibition of iNOS, and subsequently resulted in increasing of NO levels and decreasing O_2_^.-^ and nitrotyrosine levels. These effects were replicated in a cardiomyocyte biomechanical stretching diabetic model, where silencing cGCH1 blocked the preventive effect of EMPA. The beneficial effects were observed irrespective of diabetes status, although the magnitude was greater in presence of diabetes. Empagliflozin improves myocardial remodeling after myocardial infarction through overexpression of cGCH1, and irrespective of diabetes status.

## Introduction

Heart failure (HF) and atherosclerosis-related cardiovascular events represent the main causes of cardiovascular morbidity and mortality in patients with diabetes mellitus (Dm)^1^. Long-term clinical studies indicate that intensive glucose control does not prevent adverse cardiac events and rather increase HF hospitalization ^2^. In recent clinical trials, sodium-glucose co-transporter 2 inhibitors (SGTL2i) have shown to reduce significantly the risk of death and cardiovascular adverse events, including heart failure-related hospitalizations, in patients at risk with type 2 Dm^3,4^. In addition, recently, the DAPA-HF trial demonstrated for the first time that SGLT2i is able to reduce mortality and HF-associated adverse events in patients with chronic HF and reduced left ventricular ejection fraction, irrespective of diabetes status^5^. However, the exact cardiac mechanisms underlying clinical benefits, in the presence of left ventricular systolic dysfunction and heart failure, as well as the interaction with diabetes status remain unclear. Whether SGLT2i have the potential to limit post-infarct adverse ventricular remodeling is also incompletely characterized.

There is a growing interest in the role that GTP cyclohydrolase 1 (GCH1) plays in adverse cardiac remodeling^6,7^. Indeed, the presence of deficiency, inhibition or *GCH1* gene mutations result in hypertension, endothelial dysfunction, pulmonary hypertension and cardiac dysfunction^8–10^. Several reports have determined that GCH1 is the first and rate-limiting enzyme in the novo biosynthesis of tetrahydrobiopterin (BH4), an essential cofactor for all three NO synthase (NOS) isoforms. The NOS enzymes catalyze the formation of NO by oxidation of L-arginine and reduction of molecular oxygen (O_2_). In NOS catalysis, BH4 controls ‘coupling’ of the haem-oxygen intermediate to L-arginine oxidation, thus controlling the generation of either NO or superoxide (O_2_^.-^)^11^. In normal condition, when BH4 levels are normal, oxidation of L-arginine is coupled with the reduction of molecular oxygen to form NO and L-citrulline^12^. When BH4 levels become limiting, due to either a reduced biosynthesis or to oxidative loss, NOS enzymes become uncoupled and O_2_^.-^ is produced as an alternative product of the enzyme. This production of O_2_^.-^ can cause a further oxidative loss of BH4 potentiating a cardiac dysfunction and the pathogenesis of dilated myocardiopathy^13^.

This study aimed to elucidate the anti-remodeling effects of empagliflozin (EMPA) in the presence of post-MI left ventricular systolic dysfunction, the interplay with diabetes status and the myocardial mechanisms underlying, by evaluating the involvement of GCH1 and the NOS pathway. Since NOS has been involved in adverse remodeling and heart failure, this study also aims to study the relationship between these enzymes and GCH1, as potential therapeutic targets in the prevention of adverse remodeling post-MI.

## Results

### Experimental groups

**Figure 1:**
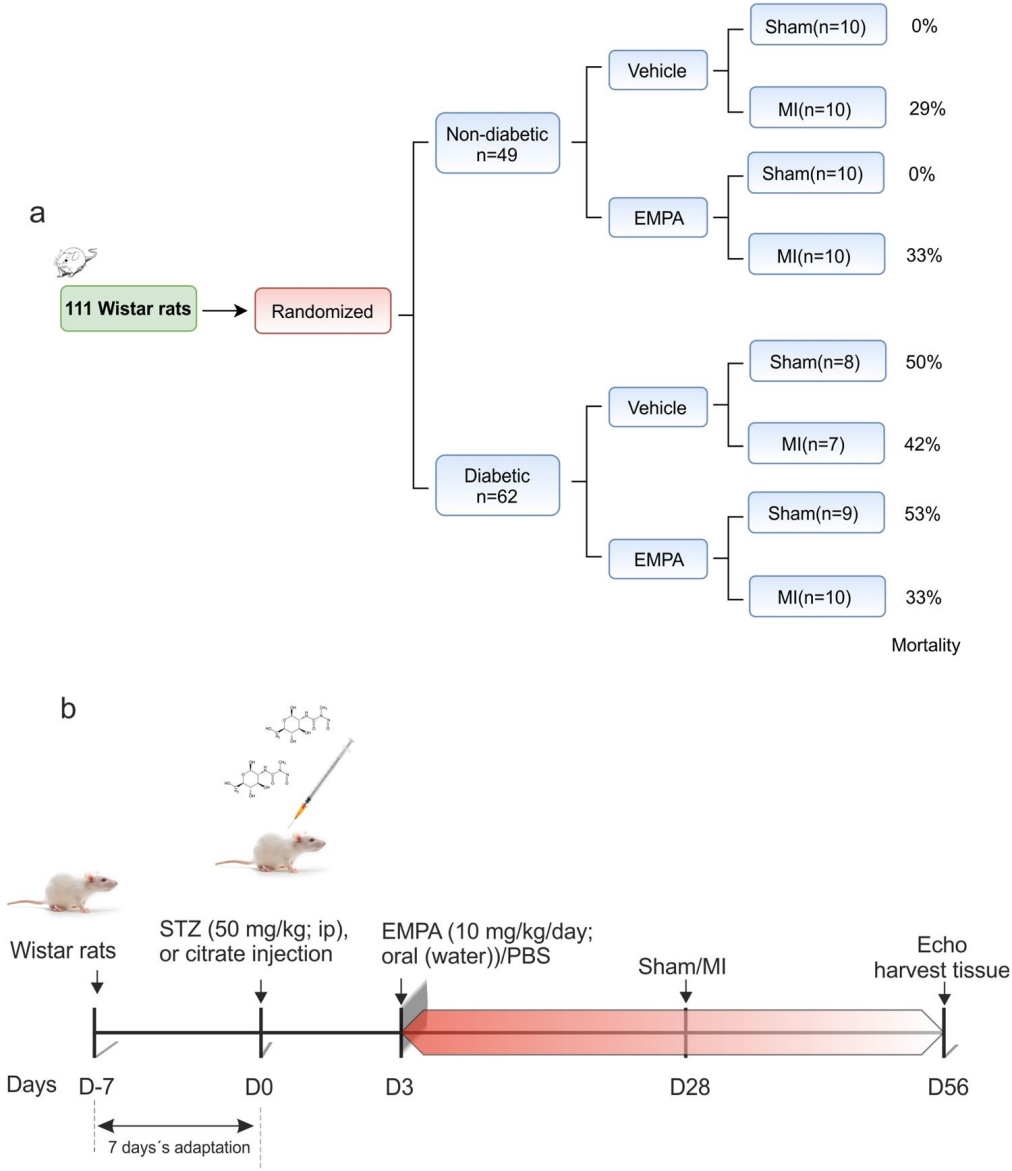
*Experimental design.* (**a**) The study consort flow diagram. (**b**) Experimental design representative scheme; the time interval where rats were treated with EMPA is shown in red. EMPA: empagliflozin; MI: myocardial infarction; STZ: streptozotocin.

The study design is presented in Fig. 1. Forty-nine non-diabetic rats and sixty-two diabetic rats were randomized consecutively to vehicle or EMPA therapy and sham or MI surgery. The procedural related mortality during was similar in all infarcted groups regardless of either diabetes status or EMPA treatment (p = 0.69). No mortality was observed during the subsequent stages of the study and no mortality was observed in sham‐operated rats throughout the study. A representative scheme of experimental protocol as showed in Fig. 1b.

### Effects on cardiac function and myocardial remodeling

**Figure 2:**
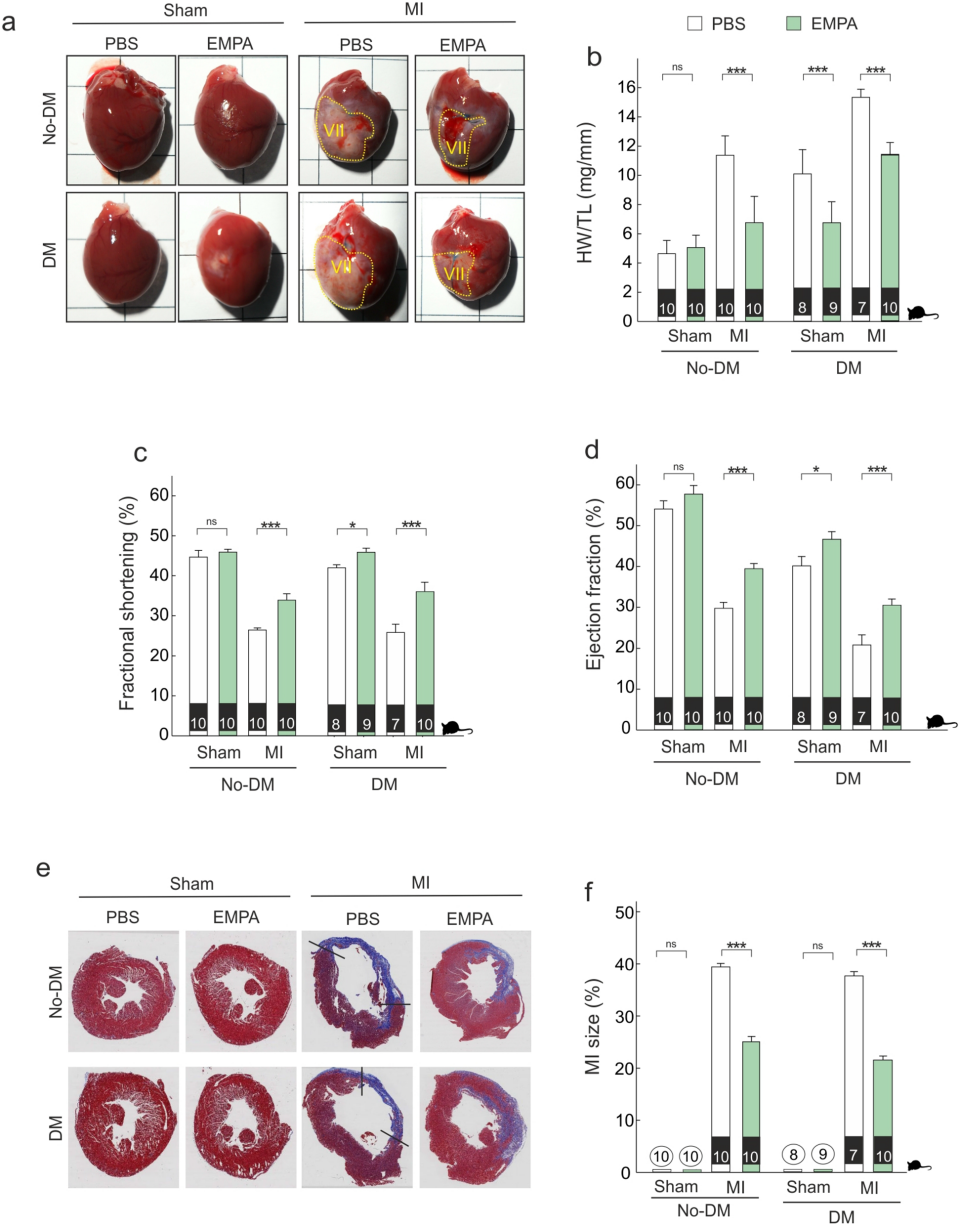
*Empagliflozin therapy improves cardiac remodeling.* (**a**) Representative images of whole heart from infarcted hearts harvested 4 weeks post-surgery. (**b**) Heart weight-to-tibia length ratio. (**c**,**d**) Echocardiographic analysis of FS and EF. (**e**,**f**) Left; Representative transversal histology sections from Masson trichrome-stained myocardium of the indicated groups taken for infarct size measurements (tissue fixation 4 weeks after MI). Collagen-rich areas (scar tissue) are colored in blue and healthy myocardium in red. Scale bar: 0.5 cm. (Right). Quantification of the infarct size. *p < 0.05, ***p < 0.001. In the lower part of each bar, number of animals analyzed per group.

The evolution of heart tissue in term of scar formation is shown in Fig. 2a. EMPA therapy was associated with lower heart weight in diabetic (diff: − 9.71 [− 11.02, − 8.37], PN > 0.999) and non-diabetic animals (diff: − 4.56 [− 5.74, − 3.34], PN > 0.999) (Fig. 2b). Following myocardial infarction (MI), EMPA therapy improved fractional shortening in non-diabetic (diff: 8.46 [5.01, 11.94], PP > 0.999) and diabetic animals (diff: 11.98 [7.98, 15.9], PP > 0.999) (Fig. 2c).

The magnitude of fractional shortening improvement did not differ between diabetic and non-diabetic animals (diff-in-diff: − 0.45 [− 2.72, 3.87]). Similar cardioprotective results were found when we analyzed the effect of EMPA therapy on other echocardiographic parameters (Table 1 and additional online Table S1) including ejection fraction (Fig. 2d). Moreover, LV infarct size was similar in diabetic vs. non-diabetic animals (40% vs. 38%) and EMPA therapy was associated with lower myocardial infarct size (Fig. 2, panels e–f) in both non-diabetic (diff: − 14.81 [− 17.11, − 12.49], PN > 0.999) and diabetic groups (diff: − 16.17 [− 18.89, − 13.58], PN > 0.999). The differential effects of EMPA in diabetic compared with non-diabetic rats are shown in additional file (Table S4).

**Table 1:** Echocardiography measurements of sham and MI control and EMPA treated non-diabetic and diabetic rats at 4 weeks post-MI.

	No-Dm				Dm			
	Sham + PBS	Sham + EMPA	MI + PBS	MI + EMPA	Sham + PBS	Sham + EMPA	MI + PBS	MI + EMPA
Nº. Animals	10	10	10	10	8	9	7	10
BW, g	314.9 ± 1.09	316.4 ± 1.89	295.5 ± 5.51	334.4 ± 4.3	204 ± 6.68	340.67 ± 5.77	199.14 ± 0.7	343.7 ± 7.25
HR, bpm	251.2 ± 2.81	250.6 ± 3.13	216.2 ± 2.64	227 ± 3.02	252.75 ± 5.87	236.11 ± 6.41	212.71 ± 4.22	245.5 ± 4.23
LVEDV, ml	0.35 ± 0.01	0.39 ± 0.01	0.46 ± 0.01	0.50 ± 0.02	0.3 ± 0.02	0.36 ± 0.01	0.67 ± 0.02	0.45 ± 0.02
LVESV, ml	0.16 ± 0.01	0.16 ± 0.01	0.33 ± 0.01	0.31 ± 0.01	0.18 ± 0.01	0.19 ± 0.01	0.53 ± 0.01	0.31 ± 0.01
SV, ml	0.46 ± 0.03	0.45 ± 0.04	0.30 ± 0.02	0.44 ± 0.01	0.54 ± 0.03	0.52 ± 0.01	0.39 ± 0.05	0.49 ± 0.06
LVEDD mm	7.75 ± 0.19	7.77 ± 0.22	10.31 ± 0.12	9.24 ± 0.16	7.77 ± 0.13	8.13 ± 0.04	10.0 ± 0.2	8.91 ± 0.22
LVESD mm	4.28 ± 0.12	4.21 ± 0.14	7.59 ± 0.11	6.00 ± 0.09	4.39 ± 0.08	4.40 ± 0.08	7.50 ± 0.25	5.64 ± 0.22
SF (%)	44.66 ± 1.67	45.91 ± 0.73	26.41 ± 0.53	34.94 ± 1.31	43.49 ± 0.68	45.87 ± 1.05	24.81 ± 2.67	36.77 ± 1.44
EF (%)	54.08 ± 2.02	57.73 ± 2.13	29.4 ± 1.46	39.48 ± 1.02	40.15 ± 2.32	46.66 ± 1.88	21.37 ± 1.9	31.49 ± 1.47

BW, body weight; EF, ejection fraction; HR, heart rate; LV, left ventricle; LVEDV, left ventricular end-diastolic volume; LVESV, left ventricular end-systolic volume; LVEDD, left ventricular dimensions at end diastole; LVESD, left ventricular dimensions at end systole; FS, fractional shortening; SV: stroke volume. Data are show as mean ± SEM. p < 0.01; p value compares mean differences between MI + EMPA and MI + PBS groups for each echocardiographic parameter.

**Figure 3:**
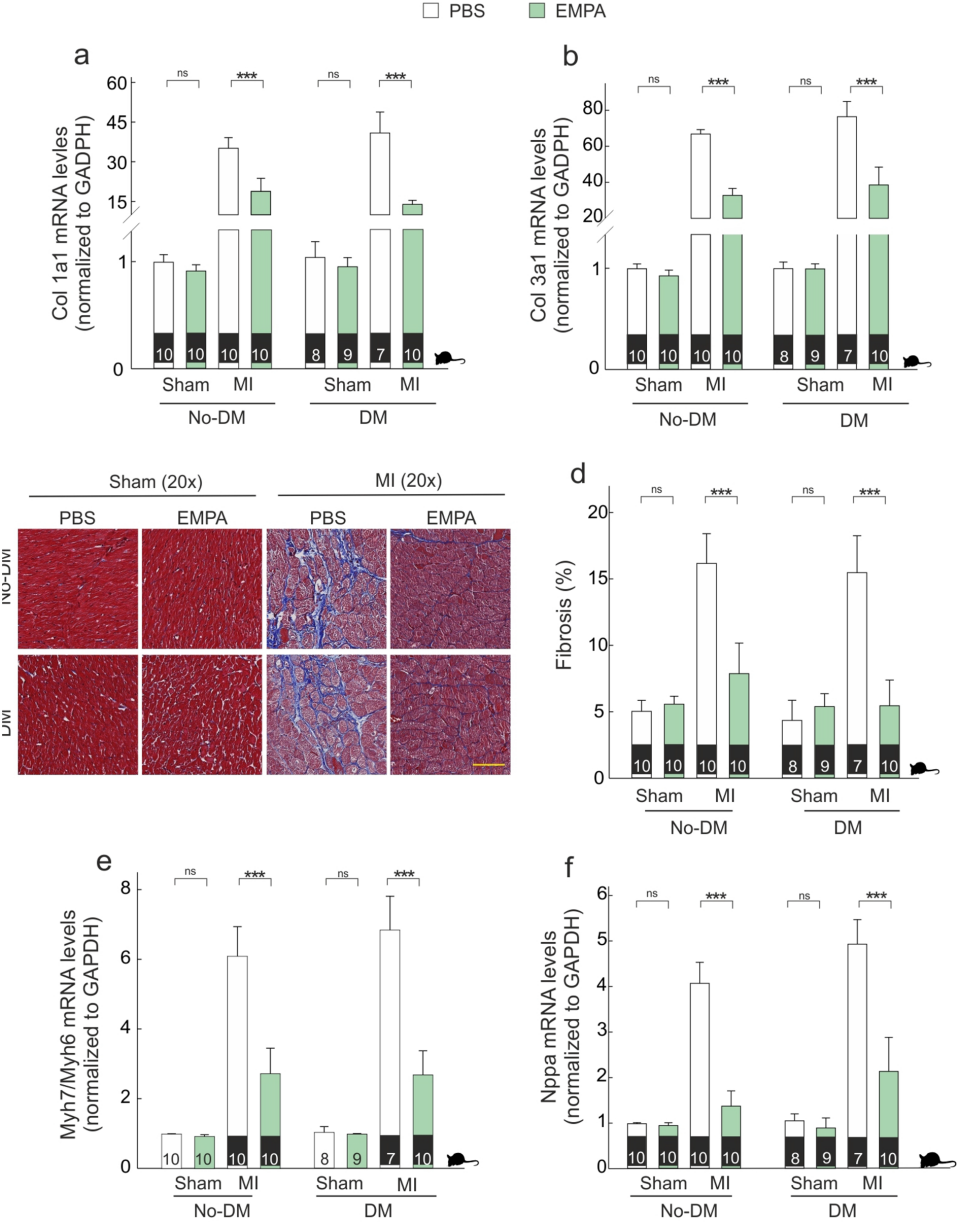
*Empagliflozin therapy prevents fibrosis and hypertrophy.* (**a**,**b**) Quantitative real-time PCR analysis of Col1a1 and Col3a1. (**c**) Representative LV sections stained with Masson; Scale bars: 25 μm. (**d**) Fibrosis-associated collagens. (**e**,**f**) Quantitative real-time PCR analysis of ratio Myh7/Myh6 and Nppa. ***p < 0.001.

Several markers of fibrosis and hypertrophy were measured in the border zone of the infarcted myocardium, and all of them were elevated following MI as compared with myocardium of sham animals (Fig. 3). EMPA therapy was associated with lower Col1a1 mRNA levels (Fig. 3a) in both non-diabetic (diff: − 16.29 [− 19.31, − 13.42], PN > 0.999) and diabetic groups (diff: − 26.9 [− 30.17, − 23.5], PN > 0.999). EMPA therapy was also associated with lower levels of Col3a1 mRNA levels in both non-diabetic (diff: − 34.06, [− 38.34, − 29.8], PN > 0.999) and diabetic animals (diff: − 37.93, [− 42.54, − 33.16], PN > 0.999) (Fig. 2b). As shown in Fig. 3c,d, EMPA therapy decreased the accumulation of collagen (Masson´s staining) in both non-diabetic (diff: − 8.24 [− 9.75, − 6.68], PN > 0.999) and diabetic infarcted animals (diff: − 9.99 [− 11.69, − 8.36], PN > 0.999). The reduction of Col1a1 was larger in the presence of diabetes (diff-in-diff: − 10.61, [− 15.17, − 5.97], PN > 0.999), whereas the reduction of Col3a1 levels (diff-in-diff: − 3.86 [− 10.2, 2.54]) and the accumulation of collagen (diff-in-diff: − 1.76 [− 4.05, 0.51]) did not differ between diabetic and non-diabetic groups.

Myh7/Myh6 ratio and Nppa mRNA levels, as hypertrophy markers, were lower in presence of EMPA treatment (Fig. 3e,f). EMPA therapy induced a lower ratio Myh7/My6 in non-diabetic (diff: − 3.37, [− 3.85, − 2.87], PN > 0.999) and diabetic animals (diff: − 4.37 [− 4.9, − 3.82], PN > 0.999).

Similar results were found on Nppa mRNA levels (Fig. 3f), with significant reductions in both non-diabetic (diff: − 2.69 [− 3.02, − 2.37], PN > 0.999) and diabetic groups (diff: − 2.9 [− 3.28, − 2.53], PN > 0.999). The magnitude of the reduction in Myh7/Myh6 ratio was larger in presence of diabetes (diff-in-diff: − 1.01 [− 1.73, − 0.29], PN = 0.995), whereas Nppa mRNA levels did not differ (diff-in-diff: − 0.21 [− 0.71, 0.28]). At overall, considering all fibrosis markers, the effect of EMPA in the diabetic infarcted groups was 35% higher (with 95% credible interval between 17 and 53%) compared to non-diabetic groups. Likewise, for hypertrophy markers, the effect of EMPA was 52% higher (95% credible interval: 30% to 81%) to that of non-diabetic groups (Additional online Figure S2).

### Effects on GCH1 enzyme, BH4 levels and NOS isoforms

**Figure 4:**
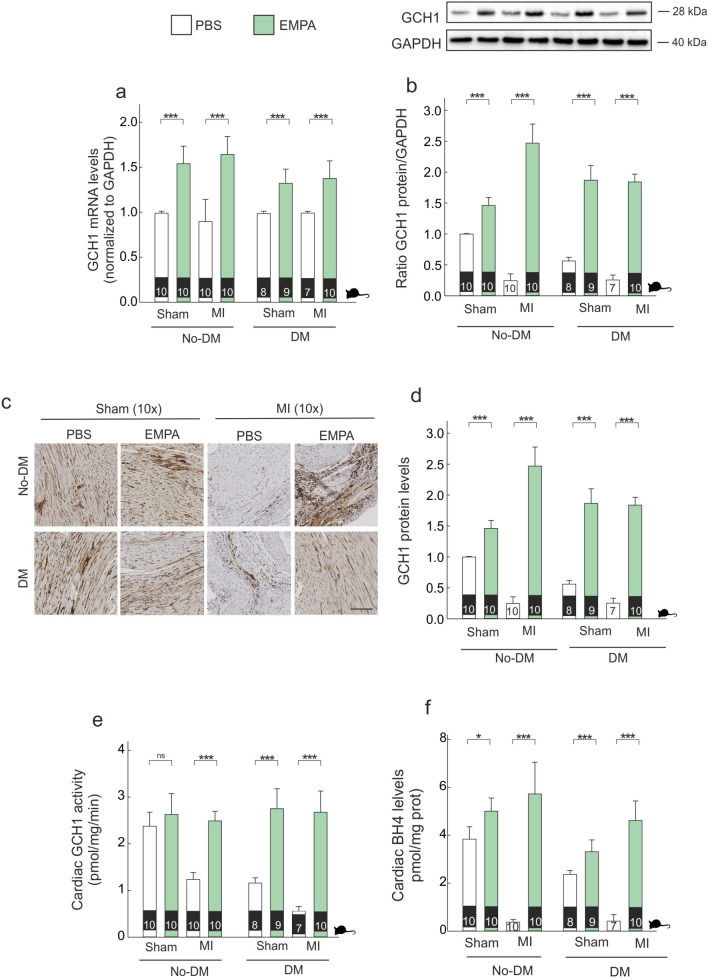
*Empagliflozin modulates mRNA and protein levels of cGCH1.* (**a**) Cardiac mRNA expression of cGCH1. (**b**) Representative western blot analysis of cGCH1 in border zone tissue and quantitative analysis. (**c**,**d**) Representative immunohistochemical cGCH1 staining in border zone; Scale bar: 50 μm. (**e**) Cardiac GCH1 activity. (**f**) Tetrahydrobiopterin levels. *p < 0.05, ***p < 0.001.

Although levels of cGCH1 mRNA did not differ in presence of either MI and/or diabetes, the expression of cGCH1 protein was lower in presence of either MI or diabetes (Fig. 4a,b). EMPA therapy induced an increase of cGCH1 mRNA and protein levels in all experimental conditions (Fig. 4a,b), which was larger in the presence of diabetes in terms of both mRNA (diff-in-diff: − 0.36 [− 0.57, − 0.14], PN > 0.999) and protein levels (diff-in-diff: − 0.63 [− 0.86, − 0.41], PN > 0.999). Cardiac GCH1 protein modulation was also confirmed by immunohistochemical staining, with similar results (Fig. 4c,d). Cardiac GCH1 activity increased with EMPA therapy, regardless of diabetic and MI status (Fig. 4e). However, the magnitude of this effect was larger in diabetic than non-diabetic animals (diff-in-diff: 0.47 [0.05, 0.88], PP > 0.986). This effect was translated in terms of cardiac BH4 levels in all experimental groups (Fig. 4f), and the magnitude of this effect was larger in non-diabetics (diff-in-diff: − 1.12 [− 1.96, − 0.25], PN = 0.994).

**Figure 5:**
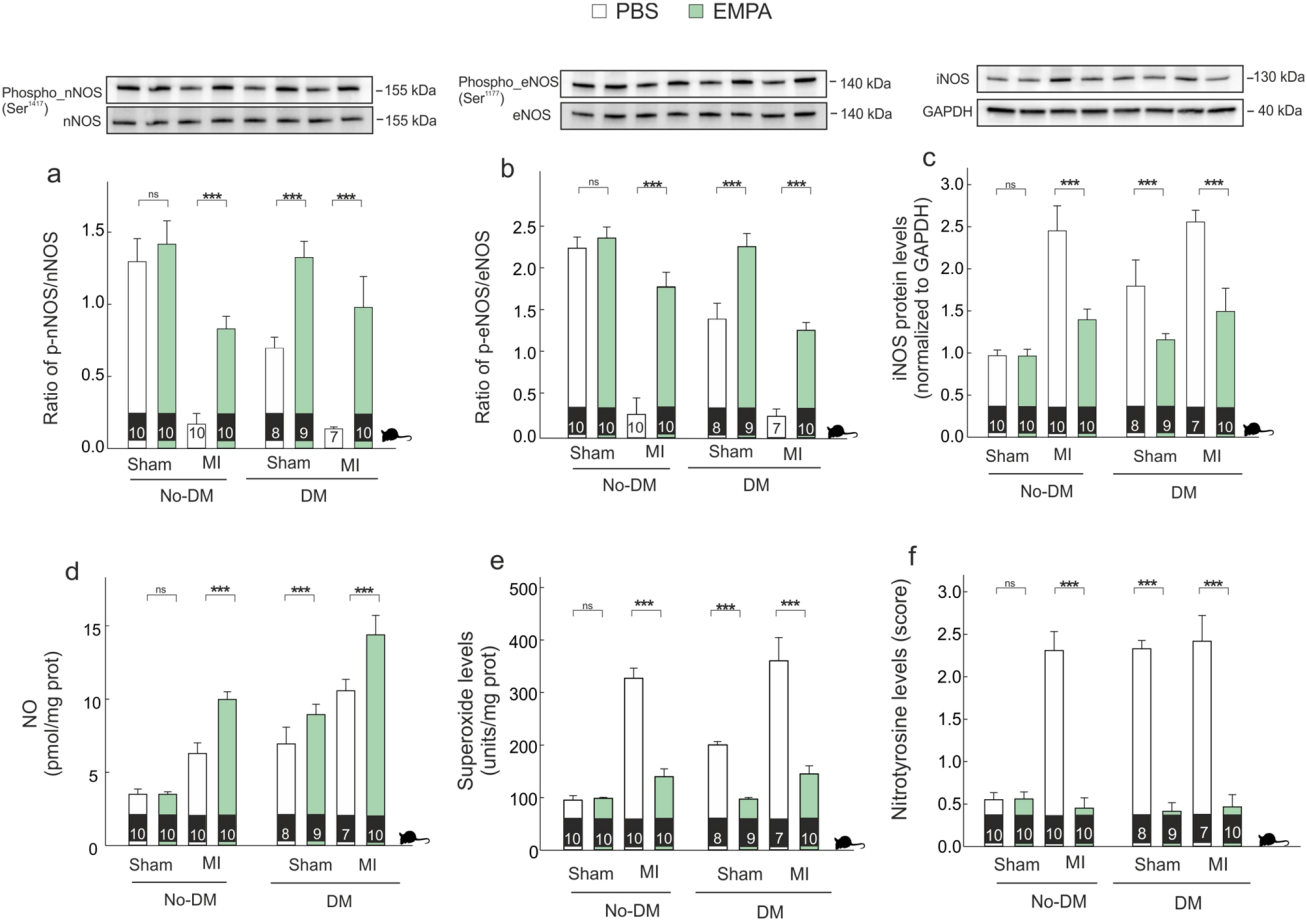
*Empagliflozin modulates NOS activity.* (**a**) Representative western blot bands showing the activation of nNOS. (**b**) Representative western blot bands showing the activation of eNOS. (**c**) Representative western blot bands showing the modulation of iNOS. (**d**) NO levels. (**e**) O_2_^.-^ levels. (**f**) Nitrotyrosine levels. ***p < 0.001.

Next, we evaluated the activation status of nNOS, eNOS and iNOS. In the presence of either diabetes or MI, the phosphorylation state of both nNOS and eNOS were lower and EMPA therapy induced an increase in both isoforms (Fig. 5a,b). This effect was greater for nNOS in diabetic than non-diabetic group (diff-in-diff: 0.2 [0.01, 0.38], PP = 0.982), whereas for eNOS the effect was greater in non- diabetic group (diff-in-diff: − 0.26 [− 0.48, − 0.05], PN = 0.991).

Regarding iNOS protein and NO levels, both increased in the presence of either diabetes or MI, and EMPA therapy was associated with lower iNOS levels and higher NO levels (Fig. 5c,d). Similar findings were observed in terms of O_2_^.-^ levels (Fig. 5e) and nitrotyrosine levels (Fig. 5f). For all these markers —iNOS protein, NO levels, O_2_^.-^ levels and nitrotyrosine levels— the effect of EMPA therapy was similar in diabetic and non-diabetic groups (diff-in-diff: − 0.02 [− 0.29, 0.24], 0.0 [− 1.13, 1.11], − 14.74 [− 38.27, 8.92], and 0.07 [− 0.16, 0.3], respectively.

### Modulation of cardiac GCH1 protein mediates anti-hypertrophic effects of empagliflozin

In order to evaluate the role of cGCH1 protein in the cardioprotective effects of EMPA, we used a pool of four small interfering RNAs to knockdown cGCH1 (siGCH1) and a diabetic biomechanical stretching model. The inhibitory efficiency of siGCH1 was verified by quantitative RT-PCR, western blot and immunofluorescence by confocal microscopy (Additional online Figure S3).

**Figure 6:**
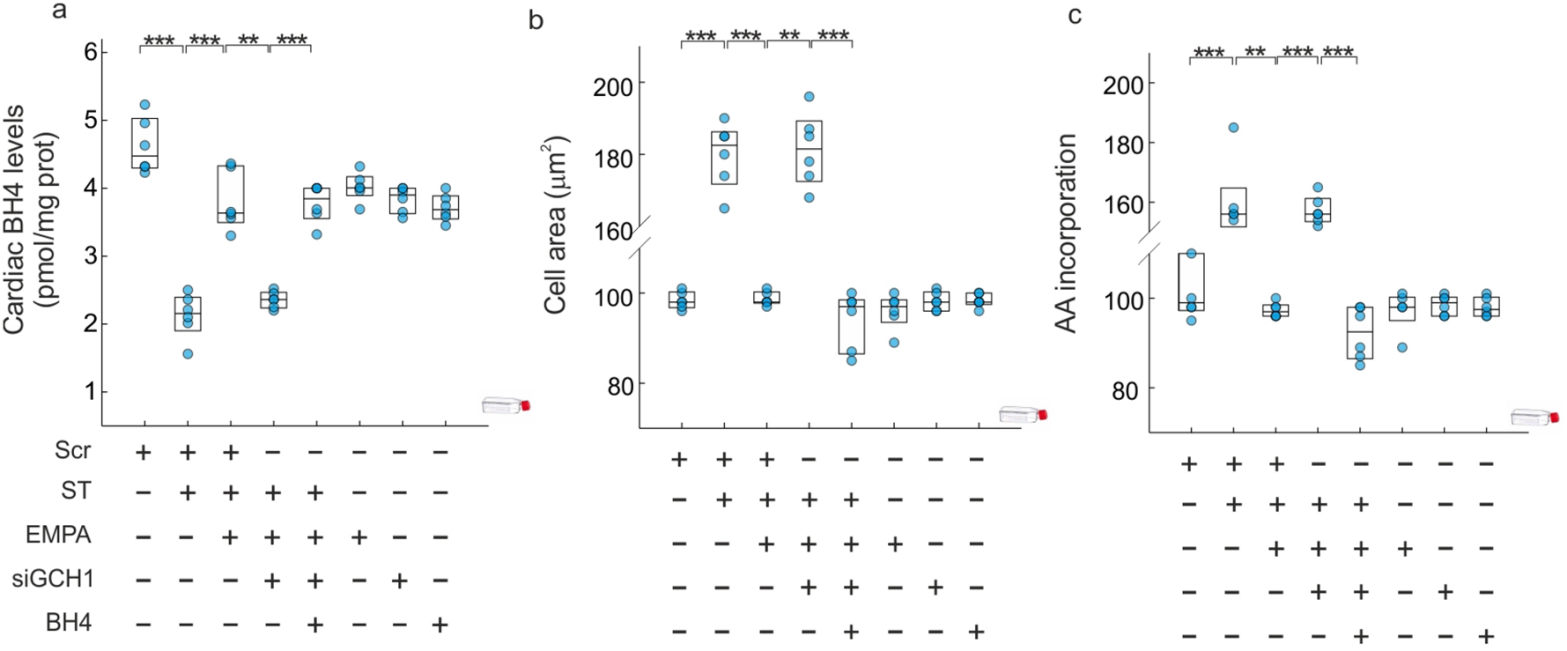
*Empagliflozin prevents cardiac hypertrophy.* (**a**) BH4 levels. (**b**) Quantitative analysis of in vitro cell size measurements of adult cardiomyocytes. (**c**) Leucine uptake in cardiomyocytes. All quantifications derive from six independent experiments/group. The results are displayed as scatter plots and summarized with boxplots. **p < 0.01, ***p < 0.001.

siGCH1 treatment blocked the preventive effect of EMPA therapy in terms of BH4 levels (diff: − 1.44 [− 1.78, − 1.11], PP > 0.999) (Fig. 6a), cell area (diff: 82.6 [76.43, 88.89], PP > 0.999) (Fig. 6b) and AA incorporation (diff: 59.7 [51.89, 67.89], PP > 0.999) (Fig. 6c). The supplementation with exogenous BH4, in presence of EMPA, reverted the action of siGCH1 in cell area (diff: − 87.24 [− 93.66, − 80.73], PP > 0.999) (Fig. 6b) and AA incorporation (diff: − 64.79 [− 72.75, − 56.65], PP > 0.999) (Fig. 6c).

**Figure 7:**
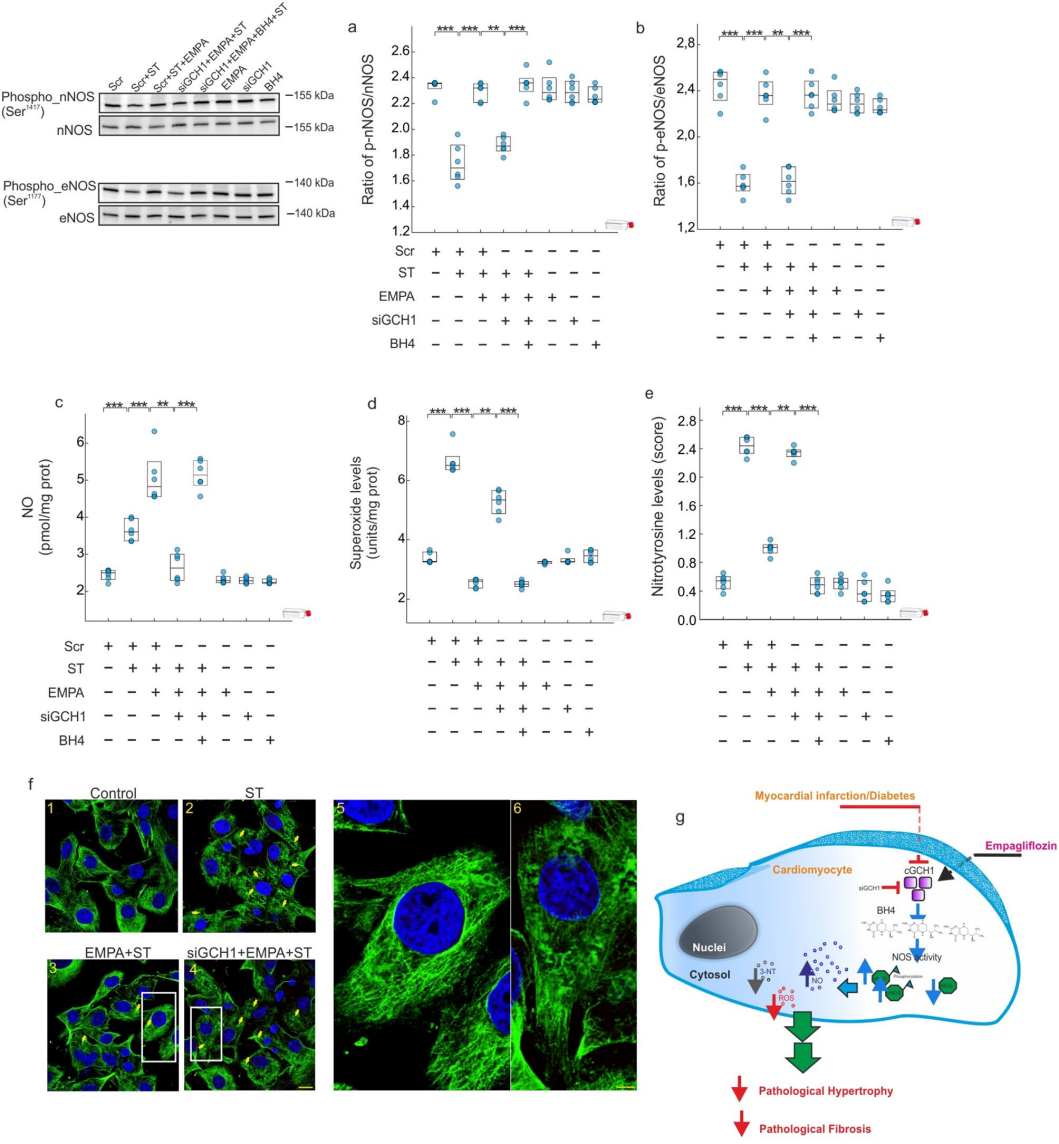
*Empagliflozin modulates NOS activity and cardiac GCH1*. (**a**,**b**) A representative western blot analysis of nNOS and eNOS activation. (**c**) NO levels. (**d**) O_2_^.-^ levels. (**e**) Nitrotyrosine levels. (**f**) Confocal microscopy images following immunofluorescence analysis performed with cardiomyocytes expressing tubulin (green). Scale bar in image 4, also applicable to 1, 2 and 3, was 40 μm whereas in image 5 and 6, was 10 μm. (**g**) Model of Empagliflozin action in the myocardium. All quantifications derive from six independent experiments/group. The results are displayed as scatter plots and summarized with boxplots. **p < 0.01, ***p < 0.001.

EMPA therapy induced an increase in the nNOS and eNOS phosphorylation in their respective activating sites Ser^1417^ and Ser^1177^ with respect to stretching (Fig. 7a,b) (diff: 0.56 [0.45, 0.66], PP > 0.999; diff: 0.77 [0.63, 0.9], PP > 0.999; respectively). siGCH1 treatment prevented both nNOS and eNOS phosphorylation induced by EMPA therapy (diff: − 0.42 [− 0.53, − 0.31], PN > 0.999;diff: − 0.73 [− 0.87, − 0.6], PN > 0.999; respectively); and both effects were reverted when BH4 was supplemented (diff: 0.47 [0.36, 0.58], PP > 0.999; diff: 0.75 [0.61, 0.89], PP > 0.999; respectively). All these effects were reproduced in parallel experiments that assessed NO (Fig. 7c), O_2_^.-^ (Fig. 7d) and nitrotyrosine levels (Fig. 7e), as measures of oxidative damage. In the absence of stretching, the use of EMPA, siGCH1 or BH4 had no effect on the evaluated parameters. Furthermore, cells exposed to high levels of mannitol (25 mM) did not show alteration of the parameters analyzed (data not shown).

Finally, siGCH1 treatment blocked the preventive effect of EMPA therapy on cardiac α-tubulin organization, which resulted in the progressive disruption of the normal architecture of microtubules, as revealed by confocal immunofluorescence microscopy (Fig. 7f).

## Discussion

The present study provides the first evidence that links the cardioprotective effects of EMPA with cGCH1 up-regulation following MI. Our results demonstrate that EMPA-induced cGCH1 up-regulation increases BH4 levels, activates both eNOS as well as nNOS enzyme and ameliorated iNOS activity, leading to an increase of available cardiac NO levels, as well as a decrease in O_2_^.-^ and nitrotyrosine levels. Together, these effects lead to an improved myocardial remodeling.

SGLT2i are anti-diabetic drugs that have exhibited marked reductions in cardiovascular events and mortality in patients with type-2 diabetes and at risk for cardiovascular events. Initially, the most clinically studied SGLT2i were EMPA^14^ and canagliflozin^15^, which have consistently reduced heart failure related outcomes in clinical trials, with further evidence suggesting a protective effects of SGLT2i on heart failure^16–19^. Recently, the evidence that another SGLT2i, dapagliflozin, prevents mortality and HF related events irrespective of diabetes in patients with HF and reduced ejection fraction has increased the interest to identify which are the cardiac molecular mechanisms underlying the clinical benefits^5^. A growing body of evidence suggests that EMPA attenuates adverse cardiac remodeling in different experimental models, such as MI induced by LAD ligation, thoracic aortic constriction or isoproterenol infusion^18,20–22^, independent of its action on SGLT2 receptors in the kidneys^20,23^. The data presented herein support the cardioprotective properties of EMPA therapy, since its long-term administration reduced the expression levels of different pro-fibrotic and pro-hypertrophic molecules, as well as scar formation and interstitial fibrosis in border zone tissue. Nonetheless, despite this growing body of evidence, neither the molecular mechanism nor the signaling pathway involved in the cardioprotective actions of EMPA is completely understood. The originality of this present work highlights the molecular basis underlying the beneficial effect of EMPA on MI-induced adverse cardiac remodeling. Furthermore, our findings extend previous observations by comparing the effect of EMPA therapy in non-diabetic vs. diabetic post-MI myocardium. We found that EMPA prevented myocardial remodeling in both contexts, although the magnitude of the effect was greater among diabetic animals (Additional online figure S2).

In a study published recently by Yurista et al., is provides evidence that both EMPA-mediated refueling of the heart as well as a decrease in oxidative damage could be related to the evaluated beneficial effects^20^ as revealed by another study published later by Iborra-Egea et al^21^. However, these studies did not focus on evaluating the signaling pathways associated with the anti-remodeling effect of EMPA therapy. The results of the current study reveal for first time a direct relationship between the pharmacological activation of cGCH1 by EMPA and the activation of BH4/NOS/NO axis, preventing the adverse cardiac remodeling following MI.

Several studies have showed as cGCH1, which is the rate-limiting enzyme in de novo synthesis of BH4, is down-regulated during adverse cardiac remodeling affecting to cardiac function^6,24,25^. In 2016 Wu et al., established that cGCH1 could be a potential therapeutic target for treatment of MI in the clinic^6^, as revealed by another study published later in showing that transgenic overexpression of cGCH1 in cardiomyocytes ameliorates cardiac remodeling following MI^26^. These authors also revealed that an transgenic overexpression of cGCH1 protein is related to a lower infarcted size^26^. In the current study, we show that cGCH1 expression is decreased in failing hearts and cardiomyocytes under stretching (Fig. 4b) which is related to a deterioration of function and increased cardiac hypertrophy (Fig. 2 and online Table S1). Furthermore, our findings demonstrate that EMPA therapy not only improved post-MI cardiac remodeling but also decreased infarct size, an effect that was associated with increased cGCH1 protein levels in the myocardium (Figs. 2 and 3). Andreadou et al., recently have showed as EMPA therapy is able to attenuate myocardial infarction in a model of diabetic rat^27^. Similarly, Lim et al., reported that a long-term oral Canagliflozin therapy attenuates myocardial infarction in both non-diabetic and diabetic rat^28^. The data presented here are in a good agreement with the above-mentioned studies. Indeed, our results demonstrate that a long-term oral EMPA therapy attenuates myocardial infarction in both non-diabetic and diabetic rat (Fig. 2e,f). Note that our results contrast with data published by Yurista et al., who reported that EMPA therapy has no effect on myocardial infarction size^20^. We believe that methodological differences may explain the discrepancies involved. More specifically, while EMPA therapy started after MI by the Yurista´s group, all of our studies are carried out with animal that were treated with EMPA four weeks before MI induction (Fig. 1b). Therefore, this difference suggest that timing and/or duration of EMPA therapy may be relevant and it must be present at the time or before of MI for cardioprotective effects.

Cumulative evidence highlights the involvement of cardiac oxidative-nitrosative stress in MI-mediated cardiac remodeling^29^. Mount et al., pointed to the key role of BH4 in NO production through an increase in eNOS and nNOS phosphorylation; leading to suppress cardiac oxidative/nitrosative stress^30^. The present study provides evidence that EMPA therapy acts as a dual activator of eNOS and nNOS (Fig. 5a,b), allowing both an increase in NO levels (Fig. 5d) as well as a decrease in O_2_^.-^ levels (Fig. 5e). A novel finding of our study is the differential effect of EMPA therapy on iNOS isoform. Ceriello et al., using an experimental diabetic model, demonstrated an increase in the *iNOS* gene expression, in parallel to the simultaneous increase of both NO and O_2_^.-^ production^31^. The data presented here are in a good agreement with the above-mentioned study. Indeed, our results demonstrate a significant increase in NO and O_2_^.-^ levels either diabetes and MI (Fig. 5d,e). Since eNOS and nNOS decreased in infarcted non-diabetic as well as in diabetic rats with or without coronary occlusion, NO derives from a significant increase in iNOS activity (Fig. 5c). Several studies have determined that iNOS-derived NO is able to induce myocardial damage^32,33^. The reaction between NO and high levels of O_2_^.-^ leads to form nitrotyrosine (Fig. 5d–f). Indeed, under elevated levels of O_2_^.-^, more NO can react and more nitrotyrosine is formed. Nitrotyrosine exerts deleterious effects, which become more relevant than the protective effect of NO. Indeed, when free nitrotyrosine was incorporated into the carboxyl terminus of α-tubulin in microtubules, altered microtubule organization and redistribution of the motor cytoplasmic protein dynein were observed^34^ (Fig. 7f). Our data determine that EMPA therapy is able to prevent these deleterious effects, by increasing BH4 levels through the activation of cGCH1, which causes an activation in eNOS and nNOS activities as well as a decreased iNOS activity. This leads to an increase in NO, decrease in O_2_^.-^ and, as expected, a significant reduction in nitrotyrosine levels. Moreover, our data suggested that this preventive effect of EMPA therapy is present after MI and it is potentiated in the presence of diabetes.

To provide further support for this argument and since the preventive effect associated with EMPA therapy, we have used a diabetic hypertrophy model in presence of high glucose concentrations. We found that the hypertrophic response in terms of cell area and AA incorporation was prevented by EMPA (Fig. 6), in agreement with the findings in the animal model. In this experimental model, we probed a direct relationship between the modulation of cGCH1 and the cardioprotective effect of EMPA therapy. First, the up-regulation of BH4 induced by EMPA therapy under hypertrophic conditions was blocked in adult cardiomyocytes lacking endogenous cGCH1. Consequently, it led to inactivation of eNOS and nNOS and a down-regulation of NO. Therefore, these findings point to the direct involvement of BH4, given that administration of exogenous BH4 reverted the negative effects observed with siGCH1 (Figs. 6 and 7).

## Conclusion

This study demonstrates that empagliflozin exerts direct anti-remodeling effects in presence of left ventricular systolic dysfunction after MI, which is irrespective of diabetes status although in a greater extent in presence of both pathological conditions. In addition, the activation of cardiac GCH1 protein was a relevant mediator of myocardial beneficial effects of EMPA, by reducing oxidative stress in the presence of MI and/or diabetes.

### Limitations

This study has several limitations, which should be pointed out. First, our conclusions are based on data obtained using an in vitro knockdown biomechanical stretching model. The next step would be to build a stronger overall evidence base using a preclinical animal model to test the modulation of this novel biomarker. Moreover, studies in the infarcted area have not been realized; where few viable myocytes coincide with other cell types. On the other hand, differences in infarct size analyzed after EMPA treatment versus no treatment may be related to new vessel formation; it´s important to analyze this cardiac effect. The GCH1 mRNA profiles differ dramatically from the GCH1 protein profiles; our results not clear why this occurs, and suggests that any effect of empagliflozin is occurring at a post-transcriptional level. In addition, data analysis and interpretation did not establish whether or not there is a direct or indirect effect of EMPA on cardiac GCH1 protein; other assays are necessary to characterize plausible intermediates.

## Material and methods

A detail description of methods is available in the online additional file

### Animals and ethics statement

The experiment protocols were approved by the University of Murcia Committee on the Ethics of Animal Experiments (Permit number: A13150105). All experiments were carried out according to current legislation. In this context, male Wistar rats (aged 7–8 weeks and weighing 250–280 g; ENVIGO RMS, SL (Barcelona, Spain)) were bred in climate-control condition with 12-h (h) light/dark cycles. Animals received standard rat chow and water ad libitum. After 7 days’ adaptation, rats were randomly split into two groups: non-diabetic and diabetic rats (Fig. 1a).

### STZ-induced diabetic model

Rats were injected intraperitoneally with streptozotocin (55 mg/kg, MERCK) dissolved in a mix of citrate buffer (citric acid and sodium citrate, pH 4.8) or vehicle (citrate buffer) (Fig. 1b). Blood glucose was checked 3 days later via tail vein; rats with a blood glucose level above 300 mg/dl were considered diabetic. Diabetic rats and non‐diabetic were then randomized to treatment with water containing EMPA (10 mg/kg/day) or control water, respectively. Rats were housed two per cage; drug dosage was calculated (each two days) by the amount of drunk water and body weight. When is indicated, EMPA therapy was maintained 4 weeks prior to MI induction and then was prolonged for another 4 weeks, prior to sacrifice of the animals (Fig. 1b). Analysis of plasma and urine parameters of diabetic and control animals treated or not with EMPA, are shown in supplementary material (Figures S4 and S5; Tables S4-S6).

### Blood glucose measurement

Blood was obtained via puncture of tail vein. The first blood drop was discarded. Blood glucose levels were measured by a glucometer (Model NC, ref., 06870333001).

### Food and water intake, and blood and urine collection

At baseline (STZ/citrate administration previous point), three days post-STZ/citrate (EMPA treatment onset), as well as seven days, four and eight weeks after EMPA treatment onset rats were weighted and placed individually into metabolic cages for 24 h measurements of food and water intake, and urine collection. Blood samples were collected via tail immediately upon removal from metabolic cages. Plasma and urine were stored at − 80 °C for later analysis. Rats were acclimatized to the metabolic cages by placing them in for short daylight periods on two separate occasions prior to the 24 h collection.

### Blood and urine measurements

Plasma and urine glucose and urine creatinine were determined enzymatically on the Roche/Hitachi Cobas system (Roche Germany). Plasma and urine sodium and potassium were measured by ion-selective electrode methods on the Roche/Hitachi Cobas system (Roche Germany).

### Coronary artery ligation

Four weeks following EMPA therapy, the levels of blood glucose were confirmed and rats were randomized to permanent ligation of the left anterior descending coronary artery (LAD) or sham surgery under isoflurane (2.5%) inhalation anaesthesia^35^.

### Echocardiography

When the rats were anaesthetized, echocardiography was performed as described previously^35^. From the four-chamber long axis views, the end-systolic (LVESV) and end-diastolic left ventricular (LVEDV) volumes were determined by the Simpson method and the ejection fraction (EF) was determined automatically as EF (%) = LVEDV-LVESV/LVEDV X 100. At the level of the chordae tendineae of the mitral valve, left ventricular end-diastolic (LVEDD) and end-systolic (LVESD) dimension measurements were made by M-mode. Data are listed in Table I and in additional file (Table S1). The investigator analyzing the data were blinded to the treatment allocation (MJFP).

### Experimental design and study protocol

To evaluate the differences in the effect of EMPA (Jardiance) therapy on myocardial infarction with or without diabetes, analysis were realized using eight experimental groups (additional online methods) that are represented on a consort flow diagram and is showed in Fig. 1a. A representative scheme of the experimental protocol used as shown in Fig. 1b. Daily food intake was comparable between EMPA and vehicle-treated groups.

### Tissue samples and histology

At the end of experimental week 4 after LAD artery ligation, animals were sacrificed and their hearts were arrested in diastole by i.v. injection of 0.2 ml 10% potassium chloride (MERCK). The heart was excised and rinsed with ice cold DPBS before to remove the right ventricle and atria. For histopathology, mid-papillary slices of the left ventricle from seven rats of each treatment group were fixed in 4% formaldehyde up to 24 h before paraffin embedding. Masson´s trichrome staining was performed to evaluate the infarct size and the extent of fibrosis^35^. For infarct size measurement, the collagen deposition highlighted (blue) was used to define the LV scarred region. Sections were evaluated under light microscope (Nikon eclipse 80i) and the images were digitized. Infarct size was calculated as percentage of the scar length to the total LV circumference on Masson’s trichrome-stained section, as described previously^35,36^. Furthermore, in the border area of each samples cardiac fibrosis was analyzed. For its quantification, at least six random pictures were taken of each slide at 20× magnification and the collagen deposition highlighted (blue) was used to define fibrosis. Percent scar circumference was determined as total infarct circumference divided by total LV circumference × 100. For other molecular-cellular biological studies, border (peri-infarct) area was collected and stored at − 80 °C for RNA extraction and Western blot. The peri-infarct area specimens were collected at least 5 mm away from the edge of the infarct area^37^. The technicians/observers who performed the individual image or molecular-cellular biological studies were blinded to the study protocol.

In vitro* assays.* Adult cardiomyocytes isolated from C25BL6/J mice^38^ were used to determine whether the cardioprotective effect related to EMPA therapy is via an up-regulation of cGCH1. To ensure the clean preparation of isolated cardiomyocytes, the mRNA levels were evaluated for c-TnT, β-MHC, and α-actinin, as specific cardiomyocytes-specific markers, using quantitative RT-PCR (additional online figure S1). All the assays were conducted 12 h after removal of serum to induce cell quiescence. Biomechanical stretching was performed using an adaptation of the experimental design described previously^38,39^. To recreate the chemical environment of diabetes mellitus and to understand the specific role of cGCH1 in mitigation of adverse remodeling and before biomechemical strain induction, cardiomyocytes were cultured for 48 h in a medium containing 30 mM glucose, high glucose (HG), in contrast to the standard glucose concentration (NG), 5.5 mM^40–42^. Then, cells were subjected to biomechanical stretching for 15 h^38^, following the experimental procedure describe in detail in additional online methods. To exclude a hyperosmolar effect, we added identical concentrations of mannitol (30 mM; MERCK, cat # M4125) in control cultures. Finally, the monolayer cultures were washed with DPBS and the cells were removed and processed for RNA and protein extraction following our previously reported protocol^35^. When indicated, 30 min before D-glucose addition, cells were incubated with EMPA (500 nM) acquired from MERCK (AMBH324A493F AMBEED, INC)^43,44^. Moreover, the experimental protocol to silence endogenous expression of cGCH1 is detailed in additional online material.

### Quantitative real time PCR assay

Total RNA was isolated both from the border area of myocardial tissue samples as well as cardiomyocytes under stretching. RNA was purified with the RNeasy Mini Kit (Qiagen), and cDNA was prepared with the iScript cDNA Synthesis Kit (BIORAD LAB. INC., Madrid) according to the manufacturer’s recommendation. Quantitative real time polymerase chain reaction (RT-qPCR) was performed with the TB Green Premix Ex Taq II (Tli RNase H Plus) Master Mix (TAKARA BIO INC., Europe), and the primers (MERCK) are shown in additional file (Table S2).

### Western blot analysis

Left ventricular specimens from the border area or harvested un-treated and treated cells were collected, washed, and lysed with RIPA (THERMOFISHER, USA) supplemented with PMSF (MERCK, USA). The total protein was extracted and the protein concentration of the lysates was quantified by the BCA protein^45^. Protein (35 μg) was denatured, separated by SDS-PAGE electrophoresis and transferred to a polyvinylidene difluoride (PVDF) membrane (MERCK MILLIPORE, USA). The transferred membranes were blocked using 5% bovine serum albumin (BSA) in TBST and incubated with the primary antibodies summarized in additional file (Table S3).

Bands were visualized using enhanced chemiluminescent ECL (AMERSHAM ECLTM Primer Western Blotting Detection Reagent (GE HEALTHCARE, NJ, USA) (RPN2232)), using a ChemiDoc XRS + system with Image Lab software from BIO-RAD LABORATORIES (Berkeley, CA, USA).

### Statistical analyses

To determine differences in mortality, according to Cochran recommendations, Fisher’s exact test was used. Data obtained were represented as mean ± S.E.M. using bar plots with error bars. Statistical differences were evaluated by fitting linear models with interactions (determined by two-way ANOVA followed by Bonferroni’s post hoc test) and estimating marginal means. Holm correction was used for multiple comparisons. P-value < 0.05 was considered as significant. With the aim of making more complex comparisons bayesian linear regressions with interactions were fitted in all cases. To assess the effect of EMPA, differences between treated and vehicle groups were calculated. In order to compare averages increases or decreases between groups, differences-in-differences were calculated. Finally, with the purpose of assess whether the benefit of EMPA is greater in the presence of diabetes in the infarcted groups relative percentage increases have also been calculated. These percentages have been averaged to obtain an overall estimate by functional groups (hypertrophy and fibrosis). Mean, 95% bayesian credible interval (BCI) and posterior probability of being positive (PP), P(μ > 0|data), or negative (PN), P(μ < 0|data), were estimated for each variable. To draw samples from the posterior distribution, 4 Markov chains of Hamiltonian Monte Carlo was used, with 1,000 warmup iteration and 1,000 sampling iterations each one. Default weakly informative normal prior distributions was used in all cases. Convergence was diagnosed with traceplots, effective samples sizes and Rhats. All the analyses were performed using R v.3.6.0 and Stan v.2.18 with the interfaces rstan and rstanarm.

## Supplementary information

Supplementary file1

## Data Availability

All data generated or analyzed during this study are presented in this article and its Supplementary Information File, or are available from the corresponding author upon reasonable request.

## Abbreviations

AA: Amino acid
BH4: Tetrahydrobiopterin
cGCH1: Cardiac GTP cyclohydrolase I
Diff: Difference
DM: Diabetic
EMPA: Empagliflozin
HF: Heart failure
LV: Left ventricular
MI: Myocardial infarction
NO: Nitric oxide
NOS: Nitric oxide synthase
PN: Posterior probability of being negative
PP: Posterior probability of being positive
Scr: Scramble
ST: Stretching
STZ: Streptozotocin
