# Author Correction: Empagliflozin improves post-infarction cardiac remodeling through GTP enzyme cyclohydrolase 1 and irrespective of diabetes status

**DOI:** 10.1038/s41598-020-73065-5

**Published:** 2020-10-09

**Authors:** Maria del Carmen Asensio Lopez, Antonio Lax, Alvaro Hernandez Vicente, Elena Saura Guillen, Antonio Hernandez-Martinez, Maria Josefa Fernandez del Palacio, Antoni Bayes-Genis, Domingo A. Pascual Figal

**Affiliations:** 1 Biomedical Research Institute Virgen de La Arrixaca (IMIB-Arrixaca), University of Murcia, Ctra. Madrid-Cartagena S/N, 30120 Murcia, Spain; 2 Cardiology Department, IMIB-Arrixaca, University of Murcia, Hospital Virgen de La Arrixaca, Murcia, Spain; 3 Endocrinology Department, Hospital Virgen de La Arrixaca, University of Murcia, Murcia, Spain; 4 Veterinary Medicine and Surgery Department, Veterinary Teaching Hospital, University of Murcia, Murcia, Spain; 5 Heart Institute, Hospital Universitari German Trías I Puyol, CIBERCV, Badalona, Madrid, Spain; 6 Cardiology Department, IMIB-Arrixaca, University of Murcia, Hospital Virgen de la ArrixacaLAIB room 2.52, Avda. Buenavista s/n, Murcia, 30120 Spain; 7 Centro Nacional de Investigaciones Cardiovasculares (CNIC), Madrid, Spain; 8 CIBERCV, Madrid, Spain

Correction to: *Scientific Reports* 10.1038/s41598-020-70454-8, published online 11 August 2020

The original version of this Article contained a typographical error in the spelling of the author Antoni Bayes-Genis, which was incorrectly given as Andoni Bayes Genis. This has now been corrected in the PDF and HTML versions of the Article, and in the accompanying Supplementary information file.

