# Supplementary material for: Empagliflozin improves post-infarction cardiac remodeling through GTP enzyme cyclohydrolase 1 and irrespective of diabetes status
Source: Sci Rep. 2020 Aug 11;10:13553. doi: 10.1038/s41598-020-70454-8 (PMC7419540; doi:10.1038/s41598-020-70454-8)
Supplement: Supplementary file 1 — Supplementary file1 [file 41598_2020_70454_MOESM1_ESM.doc]

**Empagliflozin improves post-infarction cardiac remodeling through GTP enzyme cyclohydrolase 1 and irrespective of diabetes status.**

Maria del Carmen Asensio Lopez-PhD^1&^, Antonio Lax-PhD^1&^*, Alvaro Hernandez Vicente- BSc^2^, Elena Saura Guillen-BSc^3^, Antonio Hernandez-Martinez-MD, PhD^3^, Maria Josefa Fernandez del Palacio-PhD^4^, Andoni Bayes Genis-MD, PhD^5^, and Domingo A. Pascual Figal-MD, PhD^6^*.

1.Biomedical Research Institute Virgen de la Arrixaca (IMIB-Arrixaca), University of Murcia, Murcia, Spain.

2.Cardiology Department, Hospital Virgen de la Arrixaca, IMIB-Arrixaca, University of Murcia, Murcia, Spain.

3. Endocrinology Department, Hospital Virgen de la Arrixaca, University of Murcia, Murcia, Spain.

4. Veterinary Teaching Hospital, Veterinary Medicine and Surgery Department, University of Murcia, Murcia, Spain

5. Heart Institute, Hospital Universitari German Trías i Puyol, Badalona, Spain; CIBERCV, Madrid, Spain

6.Cardiology Department, Hospital Virgen de la Arrixaca, IMIB-Arrixaca, University of Murcia, Murcia, Spain; Centro Nacional de Investigaciones Cardiovasculares (CNIC), Madrid, España; CIBERCV, Madrid, Spain

&; the authors contributed equally.

Short running head: *Empagliflozin and cardiac GTP cyclohydrolase 1*

Address for correspondence:

1) Antonio Lax Perez, PhD

University of Murcia, Murcia, Spain. Ctra. Madrid-Cartagena s/n, 30120. Tel: +34-868885281; Fax: +34-968369662. E-mail: [alax@um.es](mailto:alax@um.es)

2) Domingo A. Pascual-Figal, PhD, MD

Cardiology Department, University of Murcia, LAIB room 2.52, Avda. Buenavista s/n, 30120, Murcia, Spain. Tel: +34-868888136. Fax: +34-968369662. E-mail: [dpascual@um.es](mailto:dpascual@um.es)

**Competing interests**

The author(s) declare no competing interests.

**Funding:** This study was supported by a grant from the Seneca Foundation-Agency of Science and Technology of the Region of Murcia (20652/JLI/18) and a grant from the Instituto de Salud Carlos III (PI19/00519). Dr. Lax is a recipient of a research contract to the Spanish System of Science, Technology and Innovation by the University of Murcia.

**MATERIAL AND METHODS EXPANDED**

*Drugs and chemicals.* Empagliflozin, SGLT2i, was acquired from BOEHRINGER INGELHEIM PHARMACEUTICALS, INC., Ridgefield, CT, USA (Jardiance). Streptozotocin (S0130), sodium citrate (71402) as well as other chemicals of analytical grade were purchased from Merck (MERCK, USA).

*Induction of diabetes mellitus (DM) in Wistar rats*. DM was induced via a single intraperitoneal injection of streptozotocin (STZ) (55 mg/kg/body weight) injection, in which the STZ was dissolved in freshly prepared 0.1 M citrate buffer (pH 4.5). The non-diabetic rats (n=49) were injected with the same amount of solvent (0.1 M citrate buffer (pH 4.5)) in absence of STZ. In order to prevent hypoglycemia in the first 24 h following STZ injection, rats (n=62) were allowed to have free access to water with 5% (w/v) glucose. Three days after STZ- injection, male Wistar rats with blood glucose levels greater than 300 mg/dl were considered as diabetics and selected for the study.

*Induction of myocardial infarction (MI).* Before the surgical procedures, animals were anesthetized with isoflurane (2.5%), before being intubated and ventilated with a 16 gauge intravenous catheter and placed in supine position over a temperature control pad. The animals were monitored by electrocardiogram (ECG) electrodes connected to the limbs through small needles inserted subcutaneously. Left-sided thoracotomy was performed by a small incision between the third and fourth intercostal spaces. The incision was expanded by a blunt ended retractor in such a manner that the lungs were avoided in the area of retraction. The pericardial sac surrounding the heart was cut open, but the heart was not exteriorized. The ligation site of the left anterior descending coronary artery (LAD) was determined 8 mm away from the origin. Using a tapered atraumatic needle, a 6-0 silk ligature was passed underneath the LAD and tied with three knots. Visible blanching and cyanosis of the anterior wall of the left ventricle and swelling of the left atrium were taken as indicative of successful ligation. The procedure was considered successful if the ECG showed ST-segment elevation and the anterior wall of the left ventricle became blanched. Ribs and muscles were closed using 6-0 vicryl dissolvable sutures, leaving a small gap to aspirate any air left in the chest cavity. The air was aspirated by an in-house tube (2 mm diameter), again without touching the lungs. At the time of closure, neomycin powder and betadine were applied to the muscle and skin stitch sites, respectively. The surgical site was dressed daily to avoid any infection and to monitor for any dehiscence of the suture site. The entire procedure was performed within 20 min of the induction of anesthesia. Sham-operated rats underwent the same procedure without any ligation. After surgery, the animals received four doses of buprenorphine (0.05 mg/kg, subcutaneous) at 8 h intervals. The sham groups underwent the same surgical procedure except that the LAD coronary artery was not occluded. Electrocardiographic monitoring was used to confirm ST segment elevation.

*Experimental design and study protocol.* To evaluate the differences in the effect of EMPA (Jardiance) therapy on myocardial infarction with or without diabetes, analysis were realized using eight experimental groups that are represented on a consort flow diagram and is showed in figure 1a. Briefly, the generated experimental groups were: Group 1: Non-diabetic (STZ) sham rats receiving PBS for 54 days; Group 2: Non-diabetic (STZ) infarcted rats receiving PBS for 54 days; Group 3: Non-diabetic (STZ) sham rats receiving EMPA (10 mg/kg/day) for 54 days; Group 4: Non-diabetic (STZ) infarcted rats receiving EMPA (10 mg/kg/day) for 54 days; Group 5: Diabetic (STZ) sham rats receiving PBS for 54 days; Group 6: Diabetic (STZ) infarcted rats receiving PBS for 54 days; Group 7: Diabetic (STZ) sham rats receiving EMPA (10 mg/kg/day) for 54 days; Group 8: Diabetic infarcted rats receiving EMPA (10 mg/kg/day) for 54 days. A representative scheme of the experimental protocol used as shown in figure 1b. Daily food intake was comparable between EMPA and vehicle-treated groups.

*Echocardiography.* All the rats underwent an echocardiographic examination 24 h and 4 weeks after MI induction (just before sacrifice), using a commercial digital ultrasound system (HD11 XE PHILIPS, Andover, Massachusetts, USA) equipped with a 4–12 MHz phased array sector transducer. The echocardiographic procedure was performed by a blinded trained investigator, following the protocol of the Echocardiography Committee of the Specialty of Cardiology of the American College of Veterinary Internal Medicine and the American Society of Echocardiography recommendations^1,2^. Before the echocardiographic procedures, animals were anesthetized with ketamine (75 mg/kg, intraperitoneal) and medetomidine (0.5 mg/kg, intraperitoneal). Standard 2D echocardiographic images of the left ventricle were obtained both in long and short axis views. By means of 2D echocardiography, signs of MI were defined as any segment with increased echogenicity and/or change in myocardial thickening or systolic movement (hypokinesia, akinesia, or dyskinesia). From the four-chamber long axis views, the end-systolic (LVESV) and end-diastolic left ventricular (LVEDV) volumes were determined by the Simpson method and the ejection fraction (EF) was determined automatically as EF = LVEDV-LVESV/LVEDVX 100. At the level of the chordae tendineae of the mitral valve, left ventricular end-diastolic (LVEDD) and end-systolic (LVESD) dimension measurements were made by M-mode. Parameters are listed in table S1.

**Table S1.** *Echocardiography measurements of sham and MI control and EMPA treated non-diabetic and diabetic rats at 4 weeks post-MI.*


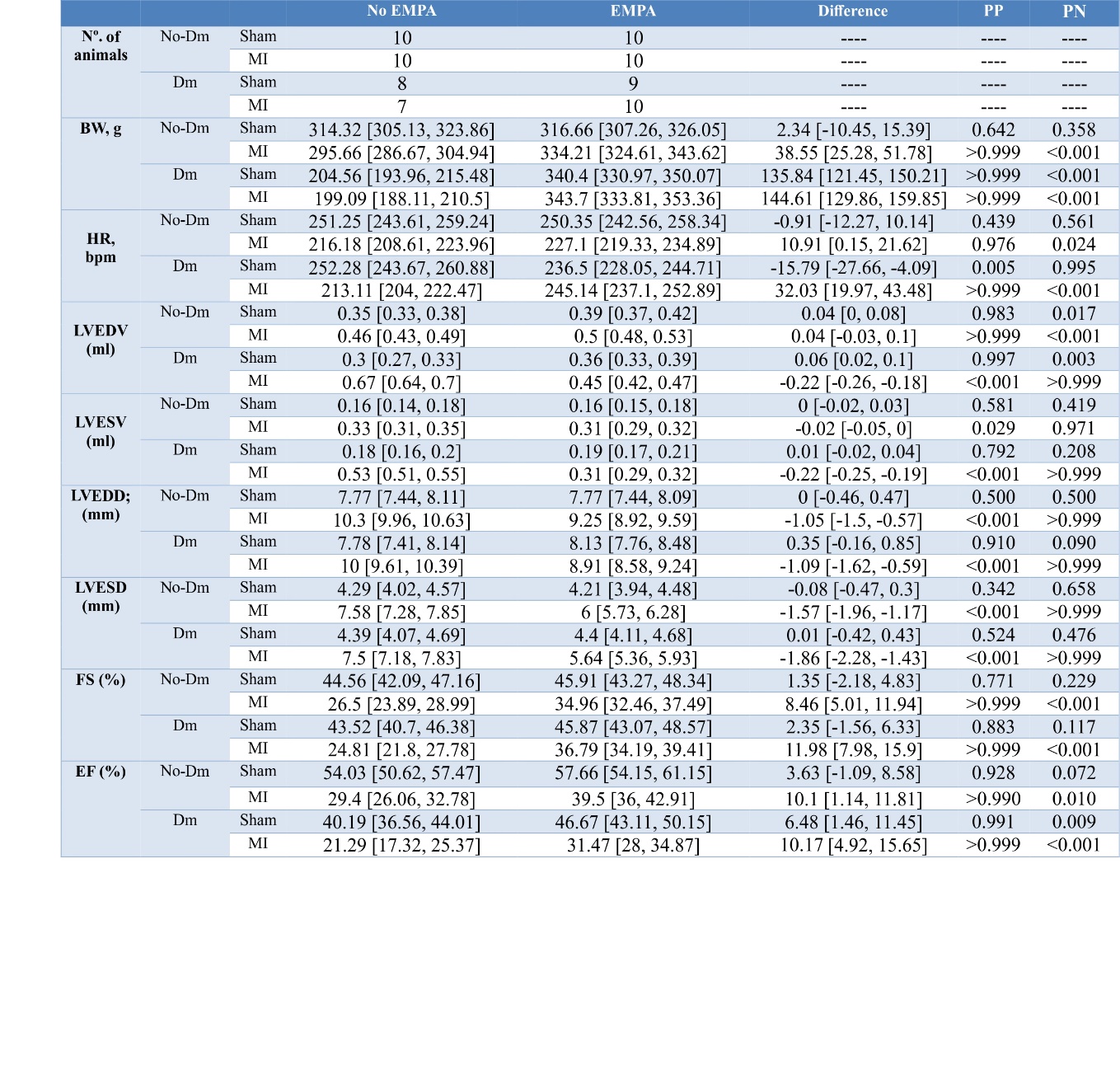
BW, body weight; HR, heart rate; LV, left ventricle; LVEDV, left ventricular end-diastolic volume; LVESV, left ventricular end-systolic volume; LVESD, left ventricular dimensions at end diastole; LVEDD, left ventricular dimensions at end systole; FS, fractional shortening; EF, ejection fraction. Data are show as posterior mean [95% credible interval]. In addition, it showed averages differences between groups. From the posterior distribution of the latter, the probability of this been positive or negative have been calculated.

*Immunohistochemistry.* Formalin-fixed and paraffin-embedded mid-papillary slice of left ventricle were sectioned (3 μm) and placed on poly-L-lysine-coated glass slides. The sections were de-paraffinized and pre-treated in DAKO PT Link for 20 min at 97 ºC using EnVision FLEX Target Retrieval Solution, High pH (DAKO, Denmark). For cGCH1 staining, rehydrated sections were incubated overnight with polyclonal antibody to GCH1 (working dilution 1:500, PROTEINTECH, Chicago, IL, USA). After primary antibody, sections were then incubated with an anti-rabbit biotinylated-labelled polymer (DAKO ENVISION,) according to the manufacturer´s protocols. Sections were finally revealed with 2-2´diaminobencidine (DAB). High-resolution images were obtained through a Leica SP8 Inverted Scanning Confocal Microscope (LEICA MICROSYSTEMS CMS GMBH, Mannheim, Germany). Six random pictures were taken of each slide at 10x magnification (n=7 slices/each treated group) and a positive immunostaining was identified as a cytoplasmic dark-brown precipitate.

*Isolation of primary cells from adult mouse.* Hearts were harvested and coronary arteries were perfused briefly with buffer A compound (113 mM NaCl, 4.7 mM KCl, 0.6 mM KH2PO4, 0.6 mM Na2HPO4, 1.2 mM MgSO4, 12 mM NaHCO3, 10 mM KHCO3, 10 mM HEPES, and 30 mM taurine) before extracting blood in a retrograde

manner by cannulating the aorta. Collagenase type II (Worthington, Lakewood, N.J.) was added to enzymatically dissociate the ventricular cells. The cells were then allowed to sediment at 37 °C for 10 min to allow the cardiomyocytes to settle as a pellet, leaving behind anon-myocyte-rich supernatant. The cardiomyocytes were then re-suspended in buffer B (47.5 mL of buffer A, 2.5 mL of FCS, and 62.5 mL of 10 mM CaCl2). CaCl2 was gradually added to the cells to a final concentration of 100 mM, and the isolated cardiomyocytes were preplated in MEM containing 5 % (v/v) FCS, 10 mM 2,3-butanedionemonoxime, 2 mM L-glutamine, and 1% (v/v) penicillin/streptomycin for 2 h at 37 °C and 5 % CO_2_.

To ensure the clean preparation of isolated cardiomyocytes, the mRNA levels were evaluated for c-TnT, β-MHC, and α-actinin, as specific cardiomyocytes-specific markers, using quantitative RT-PCR (additional online figure S1). All the assays were conducted 12 h after removal of serum to induce cell quiescence. Biomechanical stretching was performed using an adaptation of the experimental design described previously^3^. To recreate the chemical environment of diabetes mellitus and to understand the specific role of cGCH1 in mitigation of adverse remodeling and before biomechemical strain induction, cardiomyocytes were cultured for 48 h in a medium containing 30 mM glucose, high glucose (HG), in contrast to the standard glucose concentration (NG), 5.5 mM^4–6^. Then, cells were subjected to biomechanical stretching for 15 h at 37 °C with 5% CO_2_^7^. To exclude a hyperosmolar effect, we added identical concentrations of mannitol (30 mM; MERCK, cat # M4125) in control cultures. Finally, the monolayer cultures were washed with DPBS and the cells were removed and processed for RNA and protein extraction following our previously reported protocol^3^. When indicated, 30 min before D-glucose addition, cells were incubated with EMPA (500 nM) acquired from MERCK (AMBH324A493F AMBEED, INC)^8,9^.

*Biomechanical stretching model.* To induce biomechanical strain the experimental protocols described by Banerjee et al.^10^ with some modifications were followed. Briefly, primary cardiomyocytes were cultured in six-well Flex I culture plates (BF-3001C FLEXCELL INTERNATIONAL, USA) coated with collagen I. All the assays were conducted in absence of serum, as described above. The biomechanical stretch was conducted using a Flexcell FX-5000 Tension system (FLEXCELL INTERNATIONAL, McKeesport, PA). This device uses a controlled vacuum to deform the monolayer of cells grown on top of the membrane. In or case, the vacuum produced a 16% elongation on the flexible bottom elastomer membranes at a frequency of 60 cycles/min (1 Hz) for 15 h. Control cells were also plated on Bioflex plates to avoid variations based on attachment stratum and in static conditions. Knockdown models were conducted as describe later, before strain induction. In this case, cells were maintained 48 h in serum-containing medium to increase the pliability of cells to the experimental conditions. Application of a cyclic strain was associated with a significant decrease in the cellular survival index, which was similar in all assays.

*Gch1 Knockdown by RNA Interference—Gch1‐specific.* GCH1-specific siRNA or control siRNA (DHARMACON, Lafayette, CO) were trasfected into cardiomyocytes using Lipofectamine 3000, according to the manufacturer's instructions. To prevent off-target effects caused by both the sense and antisense strands, a commercial mixture of four individual duplexes designed to target one gene was used: ON-TARGET plus mouse GCH1 siRNA, previously described in^11^. Effective knockdown of GCH1 is showed in Online figure 4. Briefly, cells (~20 x10^3^) were plated into a six-well plate and grown at 37 ºC in complete medium for 2 days. Cells reaching 60 % confluence were subjected to transfection using Lipofectamin 3000 (INVITROGEN, Carlsbad, CA, USA) according to the manufacturer’s instructions. The transfection concentration was 20 nM. Cells were maintained for 8 h after adding the transfection mix, followed by recovery in serum-containing medium for another 24 h. Then, transfected cells were washed twice with DPBS at 37 ºC before biomechanical strain induction, as indicated previously. To study the effect of BH4, adults cardiomyocytes lacking in endogenous GCH1 protein were treated with 200 μM BH4 for 2 h^12^, before induction biomechanical stretching in presence of EMPA.

*Cardiomyocytes size and [3H]-Leucine incorporation assay.* To measure cardiomyocyte size, the surface area of cells was calculated using ImageJ software, selecting cardiomyocytes (~20 cells) from randomly selected fields (n=5/experiment). To evaluate the effect of [3H]-Leucine incorporation, cells were treated with 1.0 μCi/ml [3H]-Leucine (PERKINELMER) for 24 hours, washed, and fixed with 10 % trichloroacetate for 45 min at 4 °C. Then, radioactivity was determined by liquid scintillation counting (BECKMAN COULTER).

*RNA extraction and quantitative RT-PCR*. Fresh border zone tissue (~30 mg) or culture cells were washed with cold DPBS. The cells were then pelleted by centrifugation at 480 xg for 10 min at 4 ºC, while tissue samples were placed in a pre-chilled glass Petri dish and chopped in an ice bath using sharp scissors. Total RNA was isolated both from the border area of myocardial tissue samples as well as cardiomyocytes under stretching. RNA was purified with the RNeasy Mini Kit (QUIAGEN), and cDNA was prepared with the iScript™ cDNA Synthesis Kit (BIORAD LAB. INC., Madrid) according to the manufacturer’s recommendation. Quantitative real time polymerase

chain reaction (RT-qPCR) was performed with the TB Green Premix Ex Taq II (Tli RNase H Plus) Master Mix (TAKARA BIO INC., Europe). The amplified genes and primer sequences are described in online table S2.

**Table S2**. *Gene-specific primer sequences used for quantitative real-time PCR*

| Gene | Forward primer (5´→3´) | Reverse primer (5´→3´) |
| --- | --- | --- |
| Col1a1 | CATGTTCAGCTTTGTGGACCT | GCAGCTGACTTCAGGGATGT |
| Col3a1 | TCCCCTGGAATCTGTGAATC | TGAGTCGAATTGGGGAGAAT |
| GAPDH | CTGCACCACCAACTGCTTA | CAGAGGTGCCATCCAGAGTT |
| GCH1 | CCAGGTGCAGCAATGGGTTC | TTCAACCACTACCCCGACTC |
| Myh6 | GCCCAGTACCTCCGAAAGTC | GCCTTAACATACTCCTCCTTGTC |
| Myh7 | ACTGTCAACACTAAGAGGGTCA | TTGGATGATTTGATCTTCCAGGG |
| Nppa | GCTTCCAGGCCATATTGGAG | GGGGGCATGACCTCATCTT |

Col, collagen; GADPH, glyceraldehyde-3-phosphate dehydrogenase; GCH1, cardiac GTP cyclohydrolase 1; Myh, myosin heavy chain; Nppa, natriuretic peptide precursor A.

*Protein fraction.* In cold room (cold environment at 4 ºC), fresh LV (~30 mg) from border zone tissue or harvested untreated or treated cells (~ 8 x10^6^ cells), were washed using cold DPBS and gently homogenized in lysis buffer by sonication and then centrifuged at 20,000 xg at 4 °C, for 20 min. The lysis buffer was composed of: 150 mM Tris–HCl, 1 mM EGTA, 1% (v/v) Triton X-100, 1 % (w/v) sodium deoxycholate and 0.1% (w/v) SDS, pH 8.0, along with 100-fold diluted protease and phosphatase inhibitors. The resulting supernatant was aliquoted and frozen at – 80 ºC. Protein concentrations were determined by the bicinchoninic acid method^13^.

*Western blotting.* The separation and detection of proteins was performed by standard procedures, as previously described^3^. Aliquots of soluble protein were initially boiled for 6 min in SDS gel-loading buffer, and then equal amounts of protein were resolved on 8% or 10% SDS–polyacrylamide gels^3^ and electroblotted at 4 ºC on PVDF

membrane using a wet transfer system apparatus from Bio-Rad in presence of methanol for 1 h at 100 V. To evaluate high molecular weight proteins (NOS isoforms), the electroblotted experimental conditions changed to overnight at 4 ºC, 25 V and in

absence of methanol^3^. The transferred filters were blocked 1 h at room temperature with 5% BSA (w/v) in TBST (137 mM NaCl, 20 mM Tris and 0.1% (v/v) Tween-20, pH 7.6) followed by an overnight incubation at 4 ºC with primary antibody into blocking buffer. Primary antibodies and their dilutions, sources, and references, are summarized in online Table S3. Equal loading in the lanes was checked with the GADPH antibody; marker to cytosolic fraction. Then, the membranes were washed four times for 10 min each with TBST and incubated for one hour at room temperature with appropriate secondary antibodies (ECL Mouse IgG, HRP-linked whole Ab (NXA931) or ECL Rabbit IgG, HRP-linked whole Ab (NA934) from AMERSHAM BIOSCIENCES, diluted in blocking buffer at 1:10,000. After this, the membranes were washed four times for 10 min each with TBST and immunoreactive bands were detected by ECL (AMERSHAM ECLTM Primer Western Blotting Detection Reagent (GE HEALTHCARE, NJ, USA) (RPN2232), using a ChemiDoc XRS+ system with Image Lab software from BIO-RAD Laboratories (Berkeley, CA, USA). A quantitative analysis was performed with Gel-Pro Analyzer 3.1 software (Sigma). See Blue Plus2 Prestained protein standard (LC5925) from LIFE TECHNOLOGIES or Precision Plus Protein Dual Color Standars (1610374) from BIORAD were used as Prestained Protein Marker.

**Table S3**. *Summary of the primary antibodies used in the study.*

| Molecule | Dilution | Source | Reference |
| --- | --- | --- | --- |
| GCH1 | 1:500 | Abcam | ab236387 |
| Phospho-enos(Ser1177) | 1:1000 | Cell Signaling | 9570 |
| eNOS | 1.500 | Cell Signaling | 9572 |
| Phospho-nNOS(Ser1417) | 1:1000 | Abcam | ab90443 |
| nNOS | 1:500 | Cell Signaling | 4234 |
| iNOS | 1:1000 | R&D Systems | MAB9502 |
| GADPH | 1:5000 | Merck | G8795 |

*Western blot re-probing protocol.,* After film exposure, membranes were washed four times for 5 min each in TBST and incubate for 30 min at 50 °C in stripping buffer (62.5 mM Tris-HCl pH 6.8, 2 % (w/v) SDS and 700 μl β-mercaptoethanol, with slight agitation. Next, membranes were washed six times for 5 min each in TBST before blocking step.

*GCH1 activity assay*. GCH1 activity was determined as previously described with some modifications^14^. Briefly, in cold room (cold environment at 4 ºC), fresh LV (~25 mg) from border zone tissue or harvested untreated or treated cells (~ 10 x10^6^ cells), were homogenized in 50 mM Tris–HCl, pH 7.4 buffer, containing 1 mM MgCl_2_, 0.1 M KCl, 1 mM EDTA, 1 mM EGTA, freshly-added 0.2 mM PMSF, and 1.0 μg/mL each of aprotinin, leupeptin, and pepstatin. Supernatants obtained by centrifugation of samples for 15 min at 4 °C, at 12,000 ×g were transferred to a pre-chilled clean tube and centrifuged for additional 45 min at 15,000 × g. A 300 μL aliquot of the supernatant was added to 300 μL of reaction buffer: 50 mM Tris–HCl, pH 7.5, containing 0.3 M KCl and 2.5 mM EDTA; containing 2.5 mM freshly added GTP, 1 mg/ml BSA and 2.5 mM DTT. Finally, the reaction was stopped by adding 3 μl 5N HCl at 0, 1h and 2h time

points. The oxidation of the reaction product 7,8-dihydroneopterin triphosphate to neopterin triphosphate was performed at room temperature by adding 15 μL of oxidizing solution (0.04 g% iodine and 0.08 g% potassium iodide in 1 N HCl) and incubated for 60 min in the dark. The excess of unreacted I2/KI reagent was removed by the addition of 2 μl 20% ascorbic acid solution. To remove phosphate groups from neopterin triphosphate, samples pH were adjusted to 9–10 and incubated with 5U alkaline phosphatase solution (MERCK (A2356)) at 37 °C for 60 min. Samples were cleaned by filtration through filters (10K M.W.C.O MILLIPORE, Bedford, Mass.), and loaded onto a Kinetex C18 column (100mm×4.6 mm 2.6 μm, Phenomenex) and eluted with 0.05% TFA 0.7ml/min. D-Neopterin was detected by fluorescence using an excitation of 350 nm and an emission of 440 nm. Standards were used for quantification and concentrations were normalized to protein content of the samples. The protein concentration of each sample was measured using the BCA method^13^.

*Cardiac GCH1 immunofluorescence.* Cells (~ 4 ×10^5^) were plated on 35-mm glass bottomed dishes and grown at 37 ºC in complete medium for 2 days. Adherent cells were washed twice with pre-warmed DPBS, before inducing biomechanical stress. Treated and untreated cells were fixed with 4 % (w/v) formaldehyde (15 min, RT), permeabilized with 0.1% (v/v) Triton X-100 (5 min, RT) and treated with the signal enhancer kit Image-iT FX according to the MOLECULAR PROBES protocol (30 min, RT). Cells were exposed overnight at 4 ºC to anti-GCH1 (1:100, dilution). The next day, they were incubated with rabbit anti-rat IgG conjugated to Alexa Fluor 488 (1:200, dilution) for 1 h at 37 ºC. Labeled cells were viewed under a DM IRE II inverted ﬂuorescence microscope from Leica Microsystems using HCX PL APO 63x oil immersion objective with numerical aperture 1.32. Alexa Fluor 488 was excited with an argon-ion laser beam. The corresponding range of emission wavelength was 508–550 nm. Confocal images were collected with the Leica scanhead module TCS SP2. The pinhole aperture was set at 140 μm, equivalent to a z plane of 1.1 μm thickness.

*Measurement of BH4 levels.* BH4 levels were measured in border area from LV tissue homogenates or cellular samples by HPLC with electrochemical detection (ESA Biosciences CoulArray system Model 542, Chelmsford, MA, USA)(30). BH4 solutions (10–100 nM) were used as standards, and sample concentrations were normalized to protein content.

*Total nitric oxide (NO) and superoxide (O_2_^.-^) levels*. The NO concentration of tissue extracts and cell extract were determined using QuantiChrom Nitric Oxide Assay Kit (BIOASSAY SYSTEMS, Hayward, CA) according to the manufacturer's instructions. The light absorption was measured at 540 nm with use of the SpectraMAX 190 microplate spectrophotometer (GMI CO., USA). Data are expressed relative to a standard curve for NO. Lucigenin, a compound that emits light upon interaction with O_2_^.-^ was used to quantify the O_2_^.-^ production from tissue and cellular samples. The data were presented in relative light units per mg protein.

*3-Nitrotyrosine assay protocol.* 3-Nitrotyrosine (3-NT) concentration was measured using the OxiSelect Nitrotyrosine ELISA kit (STA-305, Cell Biolabs). All reagents were prepared, and the protocol was run as per the kit instructions. The samples had their absorbance read at 450 nm by a BioTek ELx800 plate reader using Gen5 software. This assay utilized competitive binding that resulted in a reverse standard curve. When analyzing the data, the standard curve was plotted on a logarithmic x-axis and utilized a

logarithmic trend line. Outlying points on the curve were removed to produce the strongest R2 value. The equation for the trend line was then used to calculate each samples respective 3-NT concentration. Each [3-NT] was then divided by the total protein content.

**Results**

*Purity of primary cells fractions.* Compared with cardiac fibroblasts, mRNA expression of c-TnT, β-MHC and α-actinin was significantly higher in primary cardiomyocytes cultures (p<0.001, for all markers) (Figure S1, a-c).


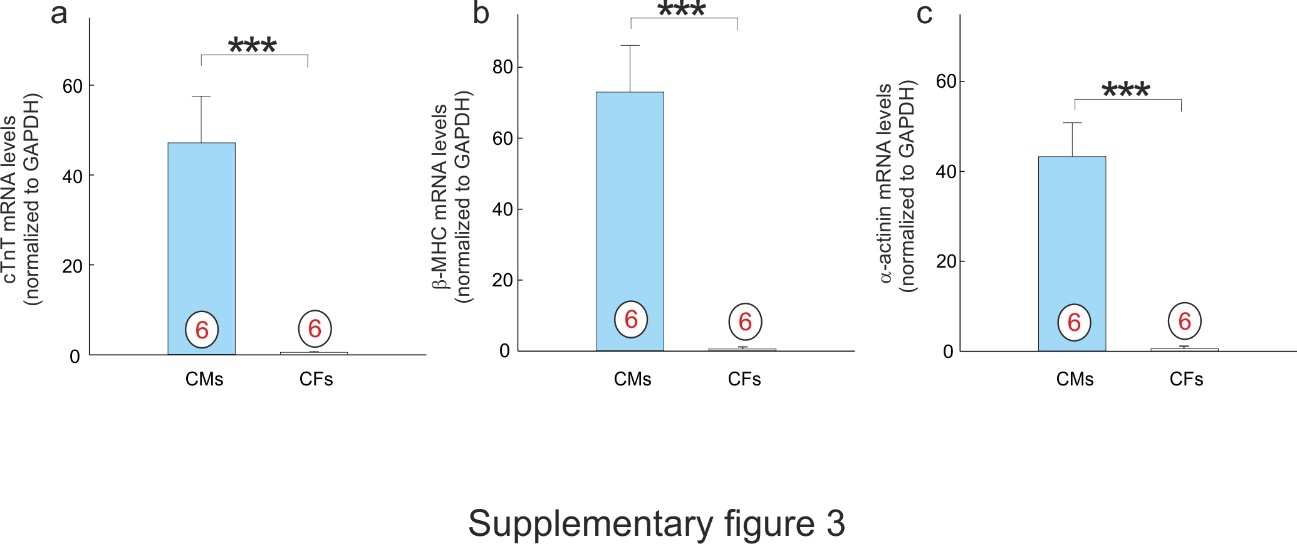


**Supplementary Figure S1**

**Purity of primary cardiomyocytes.** (a-c) Quantitative real-time PCR analysis of c-TnT, β-MHC and α-actinin from cardiac myocytes (CMs) and fibroblasts (CFs) from heart of C57BL6/J. Red numbers, represent how many independent experiments were performed per group (n=6). *** p<0.001 vs. cardiac fibroblasts. c-TnT: cardiac troponin T ; β-MHC: beta-cardiac myosin heavy chain.

*Comparative analysis of the cardioprotective effect of Empagliflozin on adverse cardiac fibrosis and hypertrophy in diabetic infarcted versus non-diabetic infarcted group.* We calculated the percentage changes with respect to the non-diabetic infarcted group (obtaining for this group the reference level of 0%) for all markers of fibrosis (Col 1a1, Col 3a1 and Collagen area) and cardiac hypertrophy (HW/TL, Myh7/Myh6 and Nppa). This, unlike the differences-in-differences, have the same scale and, therefore, allowed us to pool the estimates to get overall effects of EMPA in each functional group. Interestingly, in fibrosis this lead to obtain an average increase of 34% of EMPA effect in the presence of diabetes (Figure S2, a). Similar results were found on cardiac hypertrophy (Figure S2, b) obtaining an overall increase of 52%. These results indicate that EMPA therapy has an enhanced cardioprotective effect on diabetic infarcted myocardium in compared to non-diabetic infarcted myocardium.


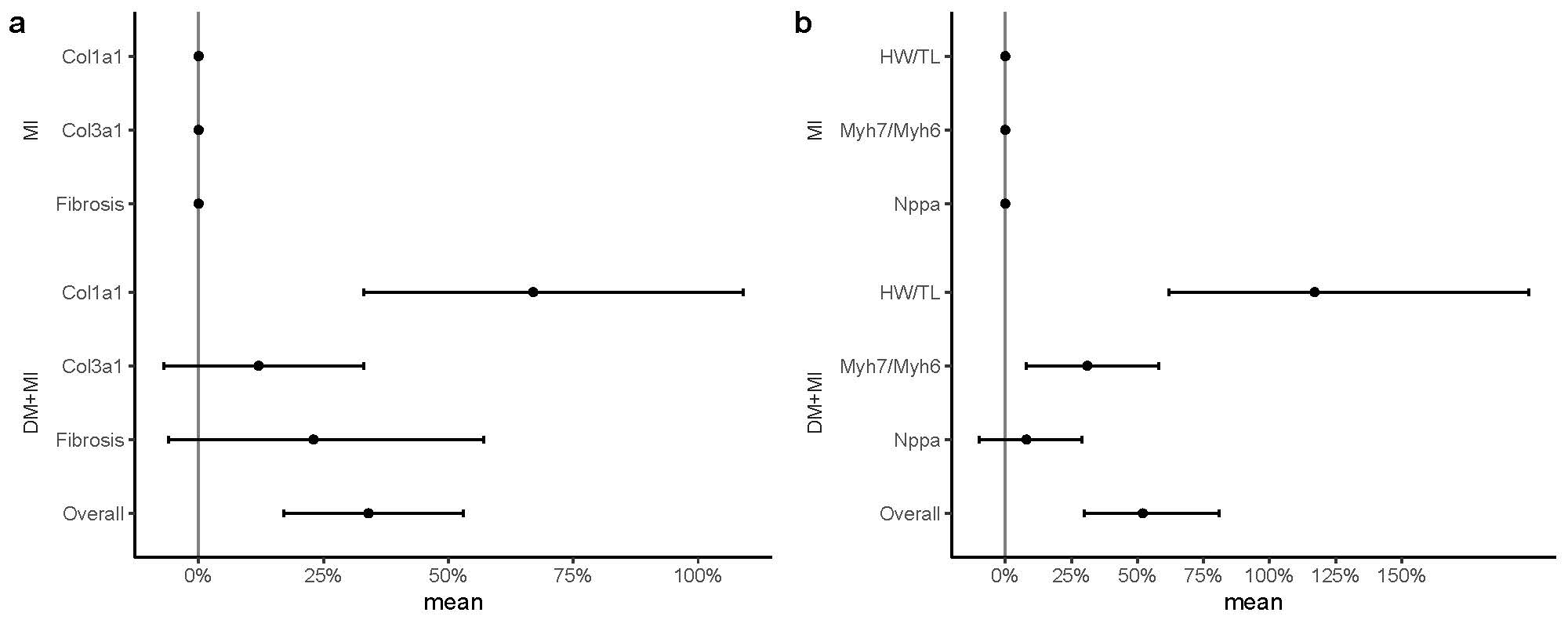


**Supplementary Figure S2**

**Cardioprotective effect of Empagliflozin and comparative statistical analysis.** Points represent mean increments in percentage in both diabetic and non-diabetic infarcted group respect non-diabetic one and error bars the 95% credible intervals. a) Fibrosis markers. b) Cardiac hypertrophy markers.

*Effective knockdown of GCH1.* As stated in material and methods section, in order to reduce the expression of GCH1, we used a pool of four target specific 19-25 nucleotide-long double stranded RNA molecules with 2-nt 3' overhangs at the end of each one (Dharmacon). As shown in figure S3 panel a, EMPA therapy induced an increase in cardiac GCH1 protein levels which was completely blocked by silencing GCH1 expression (1.96 [1.81, 2.11] vs. 1.24 [1.09, 1.39]; EMPA+ST vs. iGCH1+EMPA+ST).

Similar results were observed by immunofluorescence (Figure S3, b): the worsening of cytosolic staining of cGCH1 in cells under “biochemical stretching”, compared to the staining observed in control conditions, corroborated the fact that cGCH1 is down-regulated in response to biomechanical strain. Representative images are shown in figure S3 panel b; the green color in the images matched the cytosolic localization of GCH1. Similarly, EMPA therapy induced up-regulation of GHC1 and again the silencing of endogenous GCH1 prevented this effect mediated by EMPA therapy following strain. The effectiveness of these siRNAs, combined or single, to silence GCH1, was confirmed in cardiomyocytes by quantitative RT-PCR assays (Figure S3). The isolation of total mRNA, cDNA synthesis and quantitative RT-PCR was performed as previously described. The efficiency of each primer pair was assessed by using serial dilutions of each cDNA. Subsequently, 0.5-3 μl of template cDNA were amplified by 40 cycles of real time RT-PCR (10 s at 95 ºC, 10 s at 60 ºC and 15 s at 72 °C), followed by a dissociation stage to ensure that only one product was amplified. The EMPA-induced expression increase of GCH1 after biomechanical strain, were completely blocked when the expression of each isoform were previously silenced with the combination of the four specific siRNAs to GCH1 (p<0.001 in all cases) (Figure S3). Similar results were obtained when we pre-silenced GCH1 gene expression using single siRNAs, cited as a-d. (p<0.001).

*
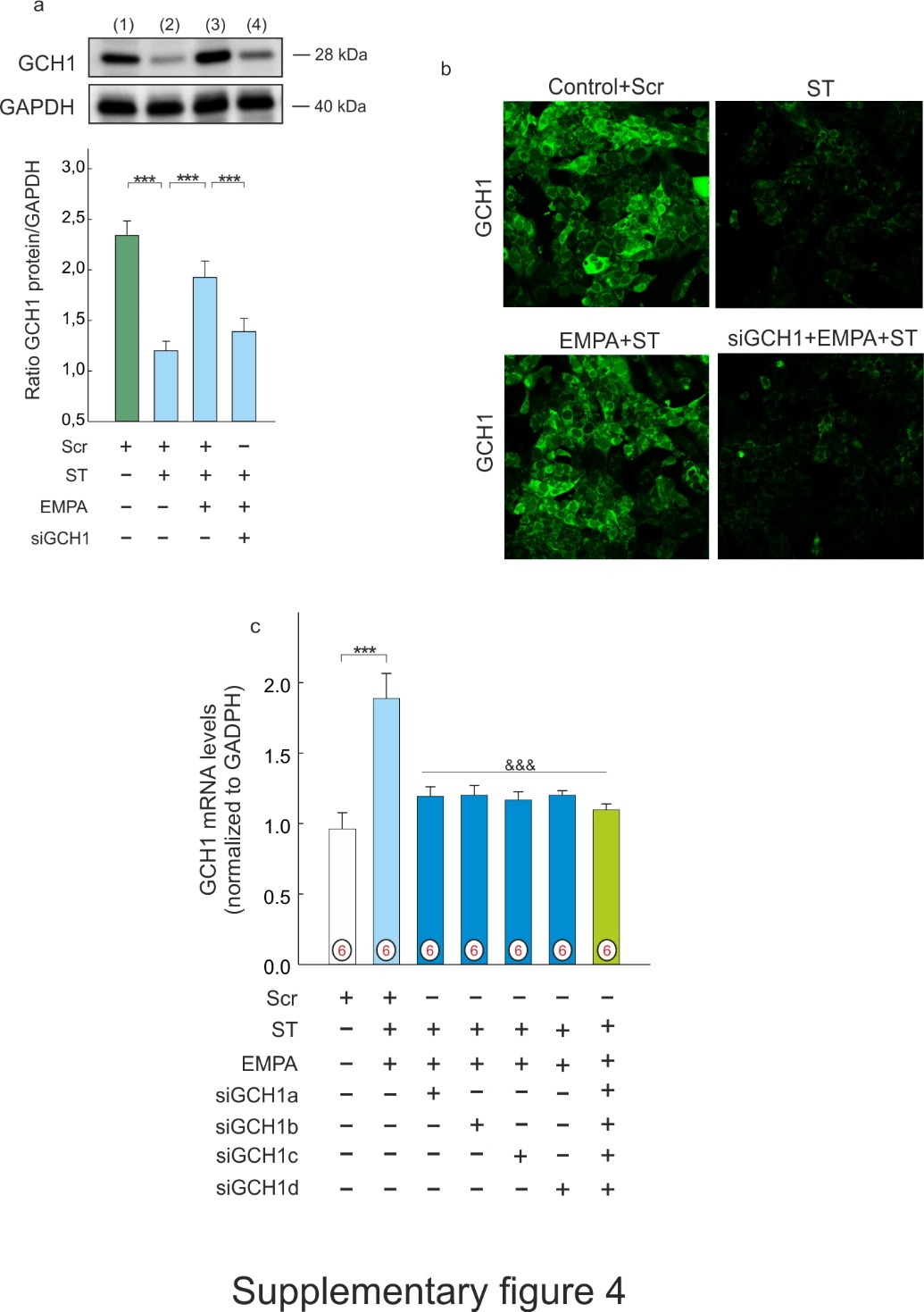
*

**Supplementary Figure S3**

**Effective knockdown of GCH1.** (a) Representative western blot analysis of GCH1; (Line 1: Scramble; Line 2: Scramble+Strain; Line 3: Scramble+Strain+EMPA; Line 4: Strain+EMPA+siGCH1). (b) Confocal microscopy images following immunofluorescence analysis performed with primary cardiomyocytes expressing GCH1 (green). (c) Cardiomyocytes were pre-incubated with scramble siRNA (scr), with a combination of four siRNAs to GCH1 (siRNA GCH1_a+b+c+d), or single siRNAs before induction of biomechanical strain for 4h. The mRNA expression of GCH1 was analyzed by quantitative RT-PCR. Red numbers, represent how many independent experiments were performed per group (n=6). Scale bar: 50 μm. ### p<0.001 vs scr; &&& p<0.001 vs Scr+EMPA+biomechanical strain. Scr: Scramble; ST: Strain.

*Effect of STZ on metabolic parameters in Wistar rats treated or not with EMPA.* Plasma parameters have indicated an increased levels of glycemia and plasma Na^+^ whereas plasma K^+^ were decreased compared to the lean non-diabetic groups, following three days STZ injection (Table S4 and blue line in figure S4). EMPA therapy significantly decreased glycemia, and Na^+^ levels whereas increased K^+^ levels (Table S4 and red line in figure S4). In addition, EMPA therapy increased urinary glucose and Na^+^ excretion (Table S5 and red line in figure S5) in parallel with an increase in urine volume and had no effect on albumin/creatinine ratio. The increase in diuresis after EMPA treatment was compensated by a proportionate increase in fluid intake (Table S6 and red line in figure S5).


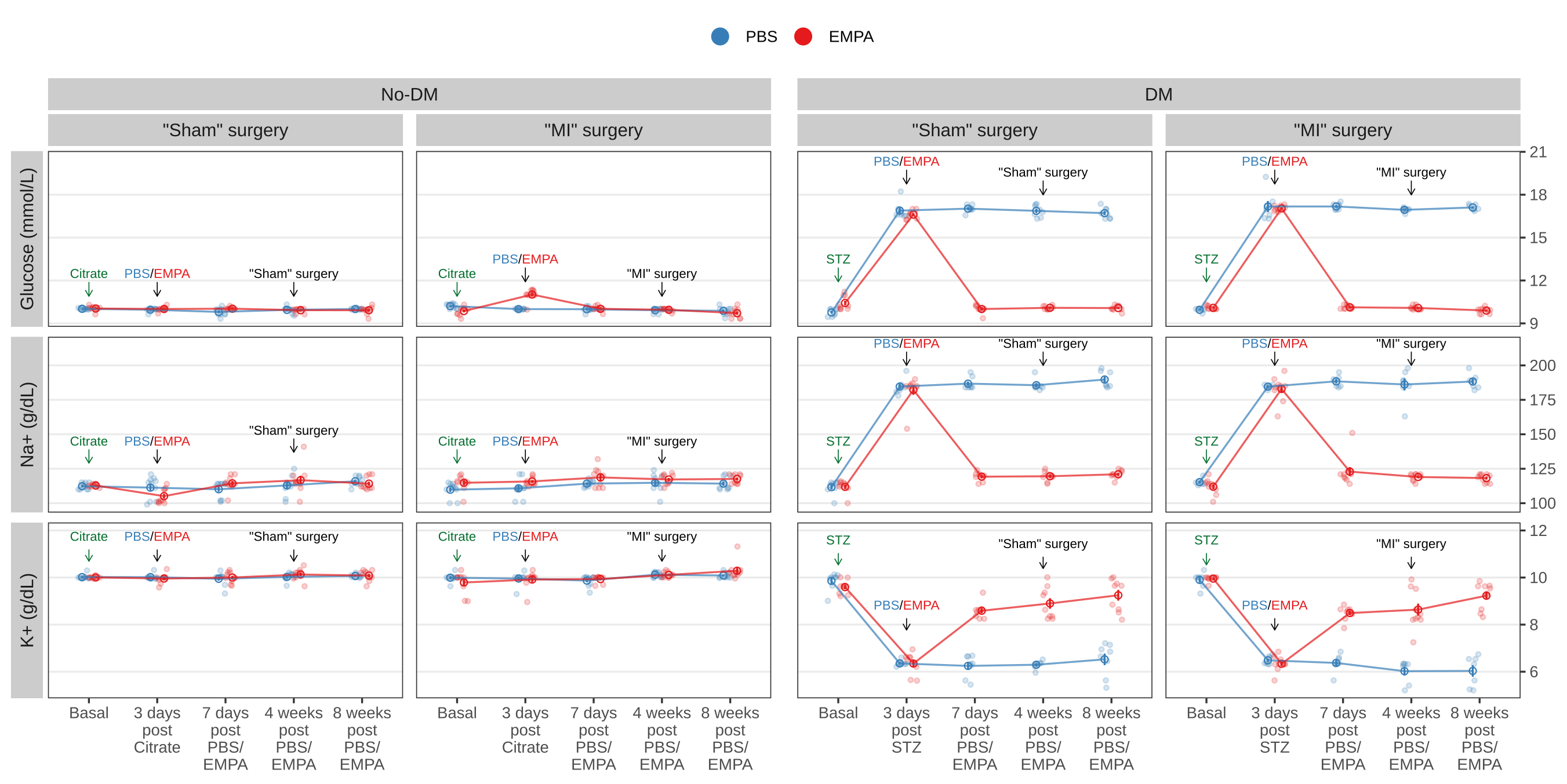


**Supplementary Figure S4**

**Effect of STZ and EMPA therapy on plasma parameters.**

**
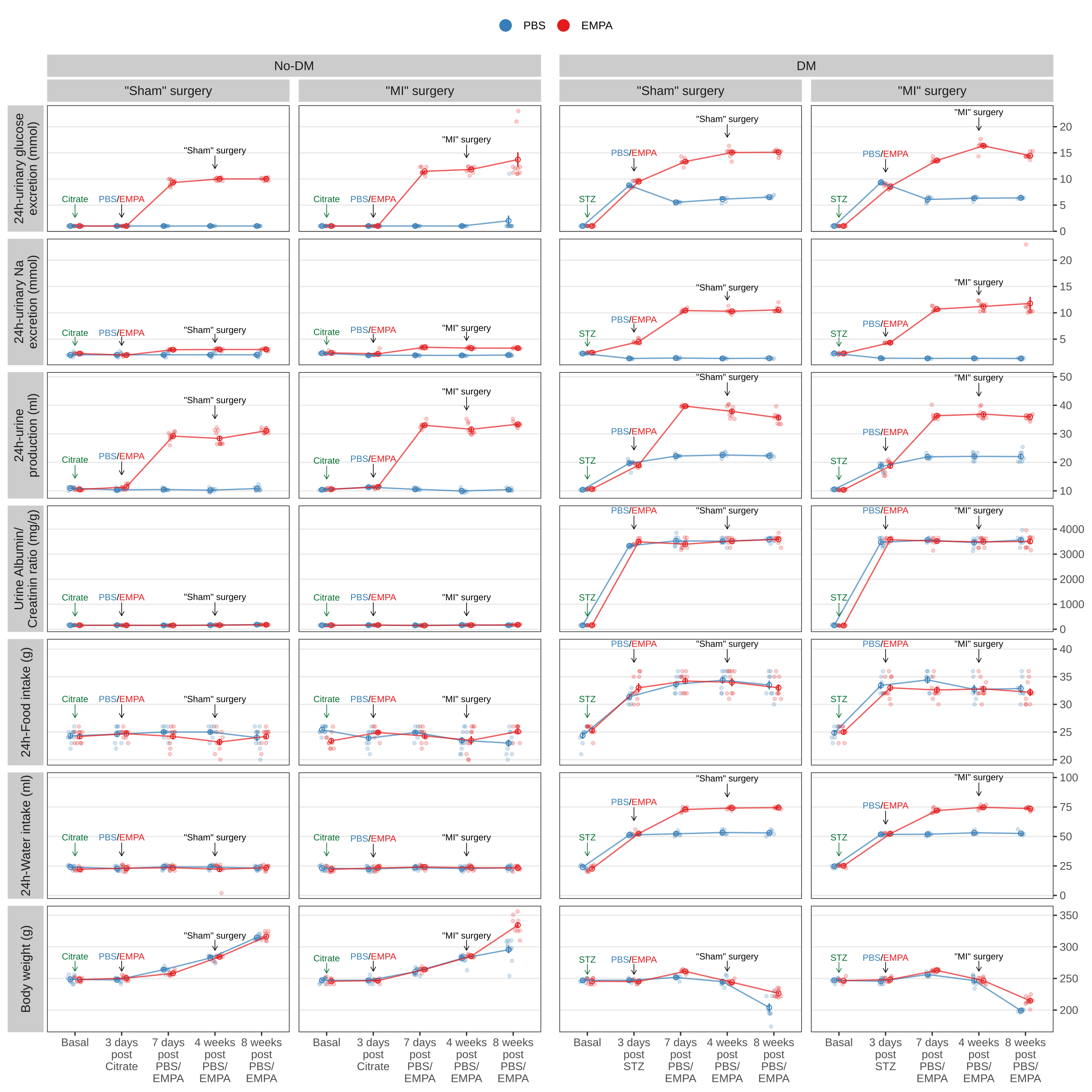
**

**Supplementary Figure S5.**

**Effect of STZ and EMPA therapy on urine parameters and intake parameters**


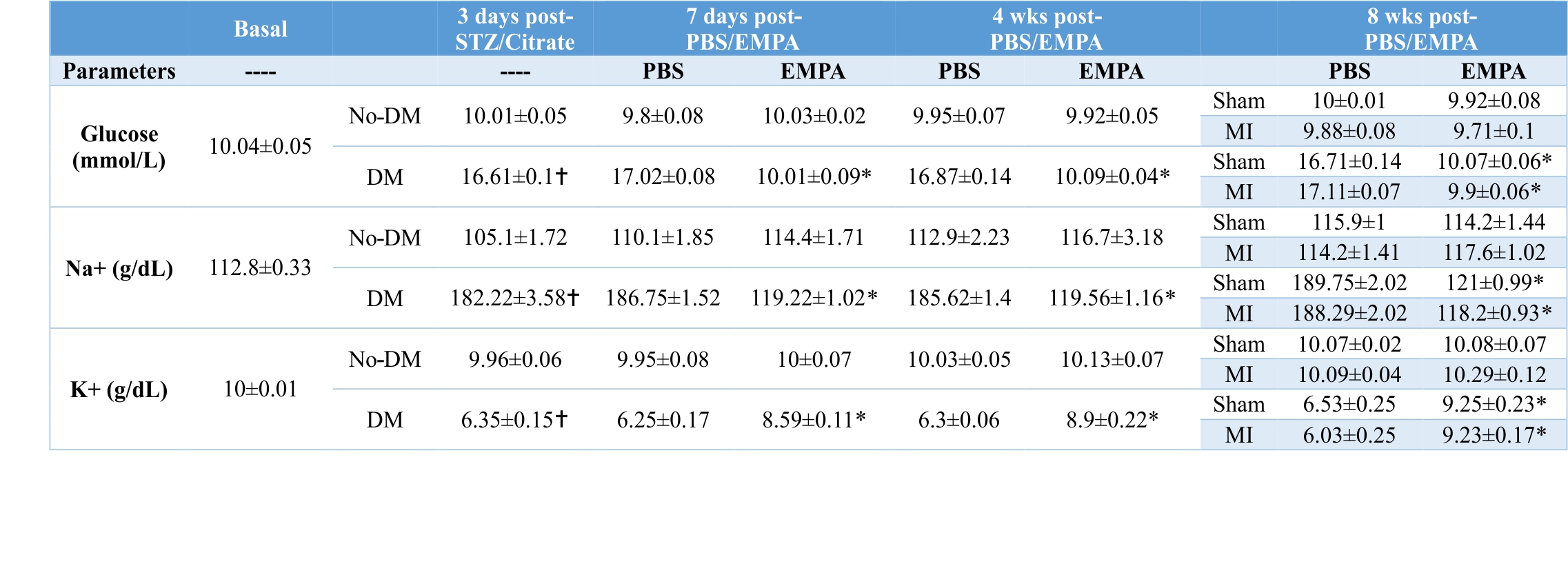
Online table S4. Effect of STZ and EMPA therapy on plasma parameters.

EMPA: empagliflozin; STZ: streptozotocin. Data are shown as mean±SEM. ✝ p<0.01; p value compares mean differences between STZ/Citrate group and its basal group, for each parameter; * p<0.01, p value compares mean differences between PBS/EMPA group and its basal group, for each parameter.


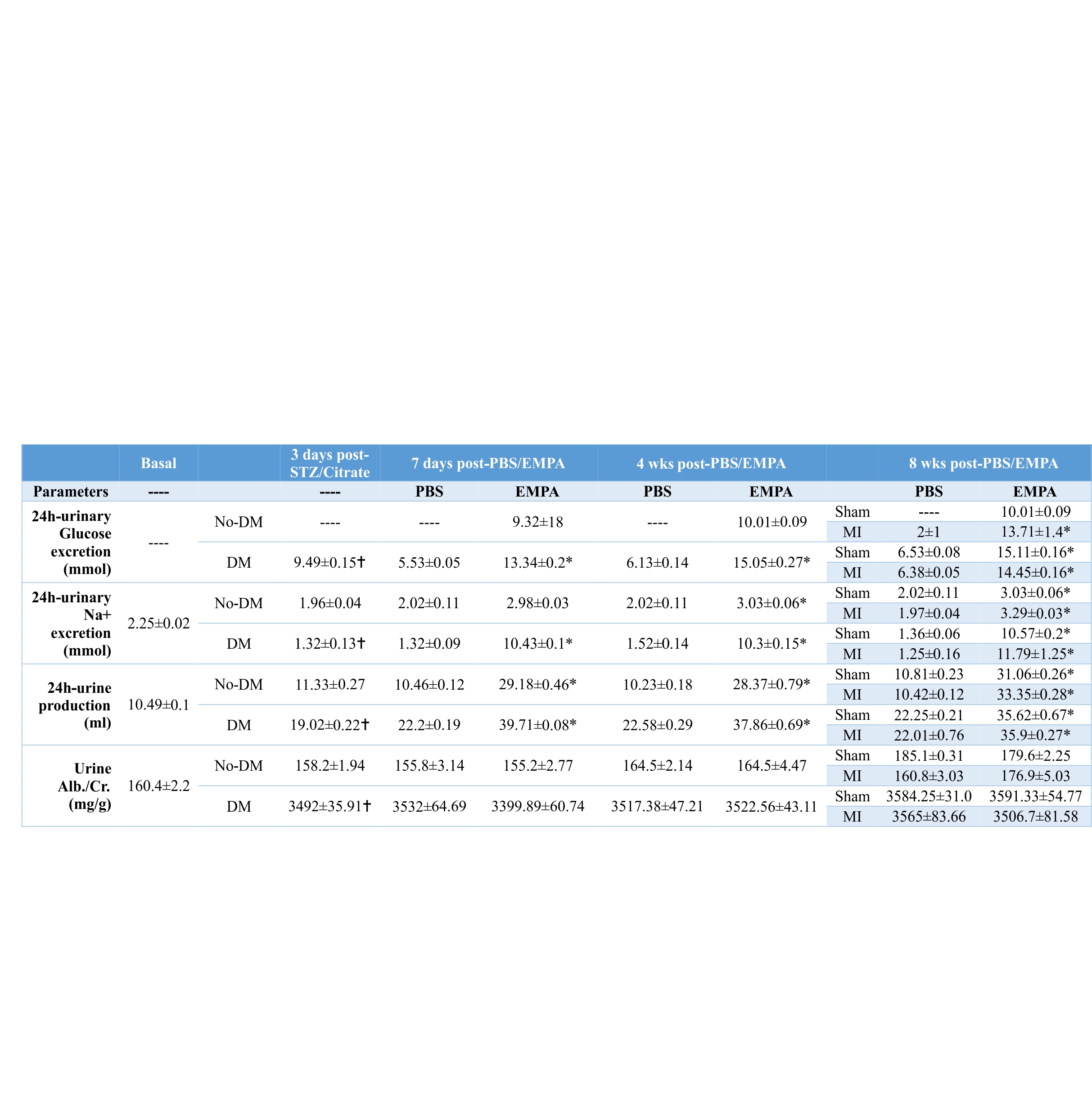
Online table S5. Effect of STZ and EMPA therapy on urine parameters.

EMPA: empagliflozin; STZ: streptozotocin. Data are shown as mean±SEM. ✝ p<0.01; p value compares mean differences between STZ/Citrate group and its basal group, for each parameter; * p<0.01, p value compares mean differences between PBS/EMPA group and its basal group, for each parameter.


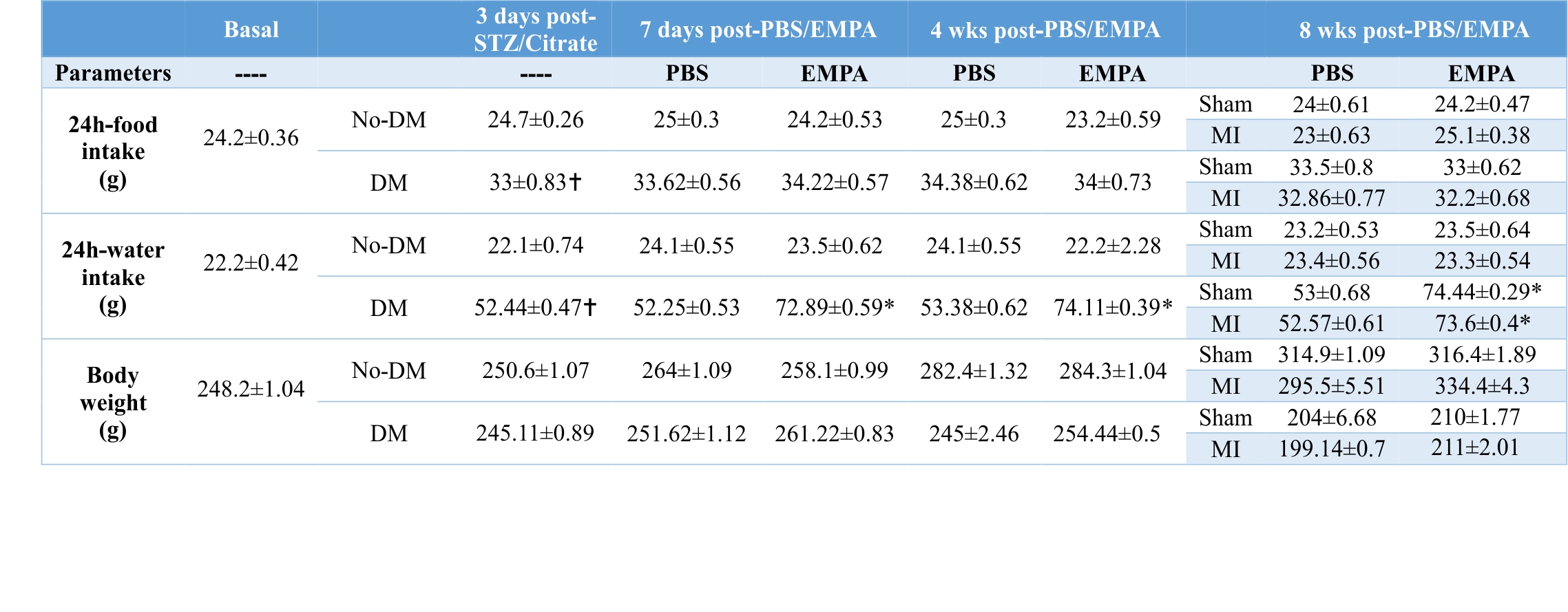
Online table S6. Effect of STZ and EMPA therapy on intake parameters

EMPA: empagliflozin; STZ: streptozotocin. Data are shown as mean±SEM. ✝ p<0.01; p value compares mean differences between STZ/Citrate group and its basal group, for each parameter; * p<0.01, p value compares mean differences between PBS/EMPA group and its basal group, for each parameter.


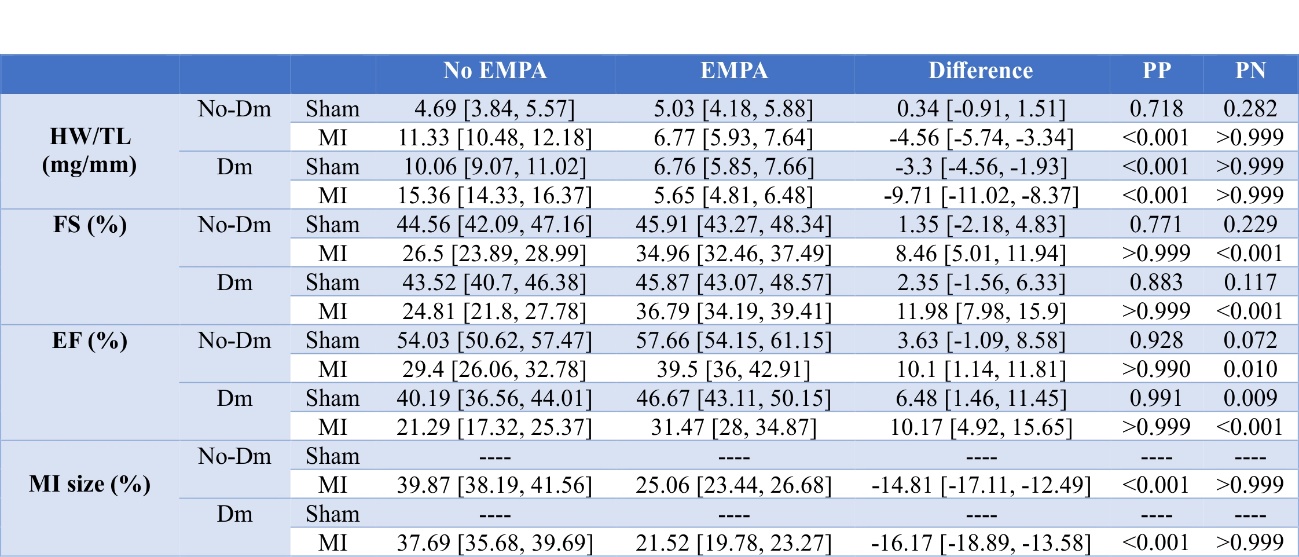
Online table S7. Results respect to figure 2 (Main Manuscript).

EF, ejection fraction; HW, heart weight; MI myocardial infarction; TL, tibia; FS, fractional

shorting. Data have been represented as described above, in table S1.


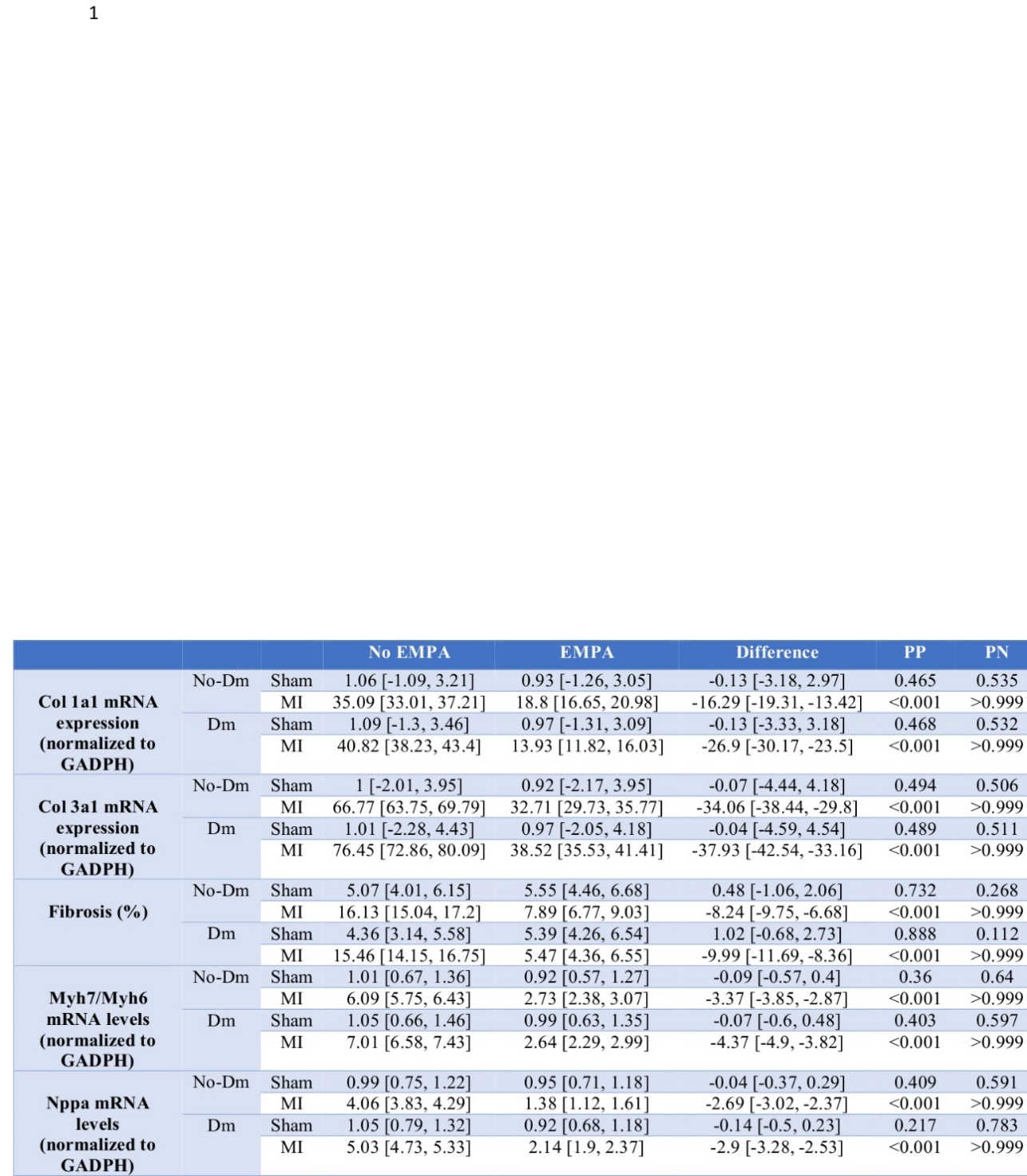
Online table S8. Results respect to figure 3 (Main Manuscript).

Col, collagen; GADPH, glyceraldehyde-3-phosphate dehydrogenase; Myh, myosin heavy chain; Nppa, natriuretic peptide precursor A. Data have been represented as described above, in table S1.

Online table S9. Results respect to figure 4 (Main Manuscript).


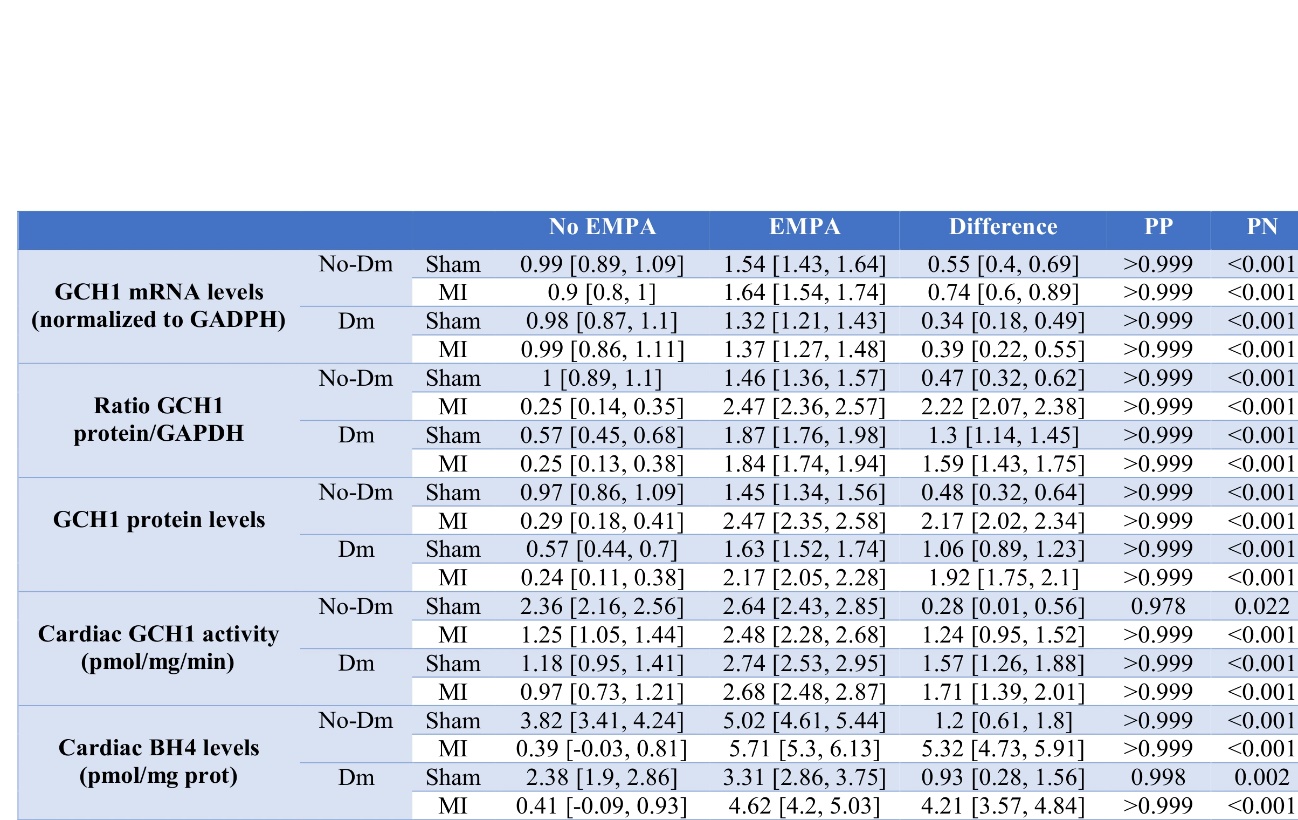
BH4, tetrahydrobiopterin; GADPH, glyceraldehyde-3-phosphate dehydrogenase; GCH1, cardiac GTP cyclohydrolase 1. Data have been represented as described above, in table S1.


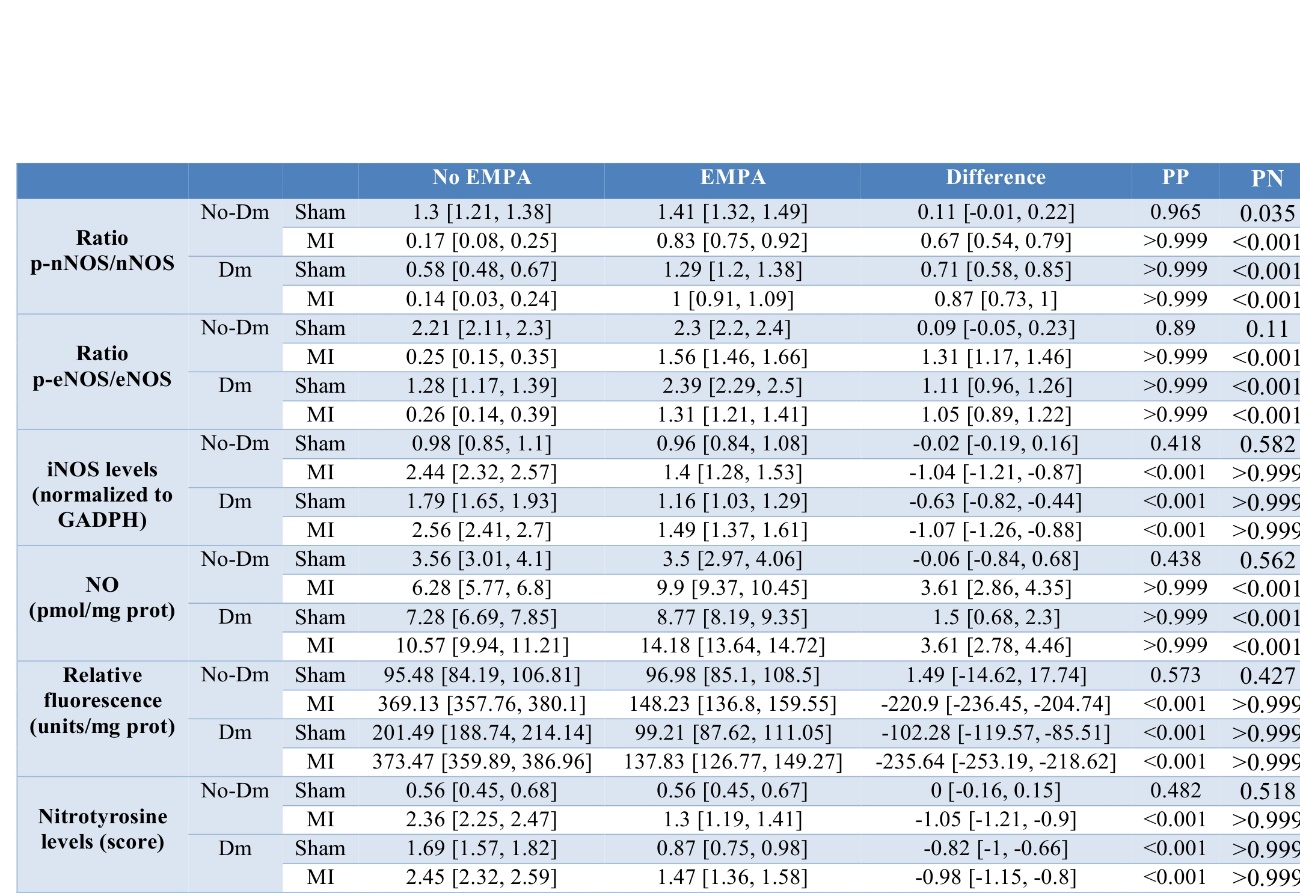
Online table S10. Results respect to figure 5 (Main Manuscript).

NO, nitric oxide. Data have been represented as described above, in table S1.


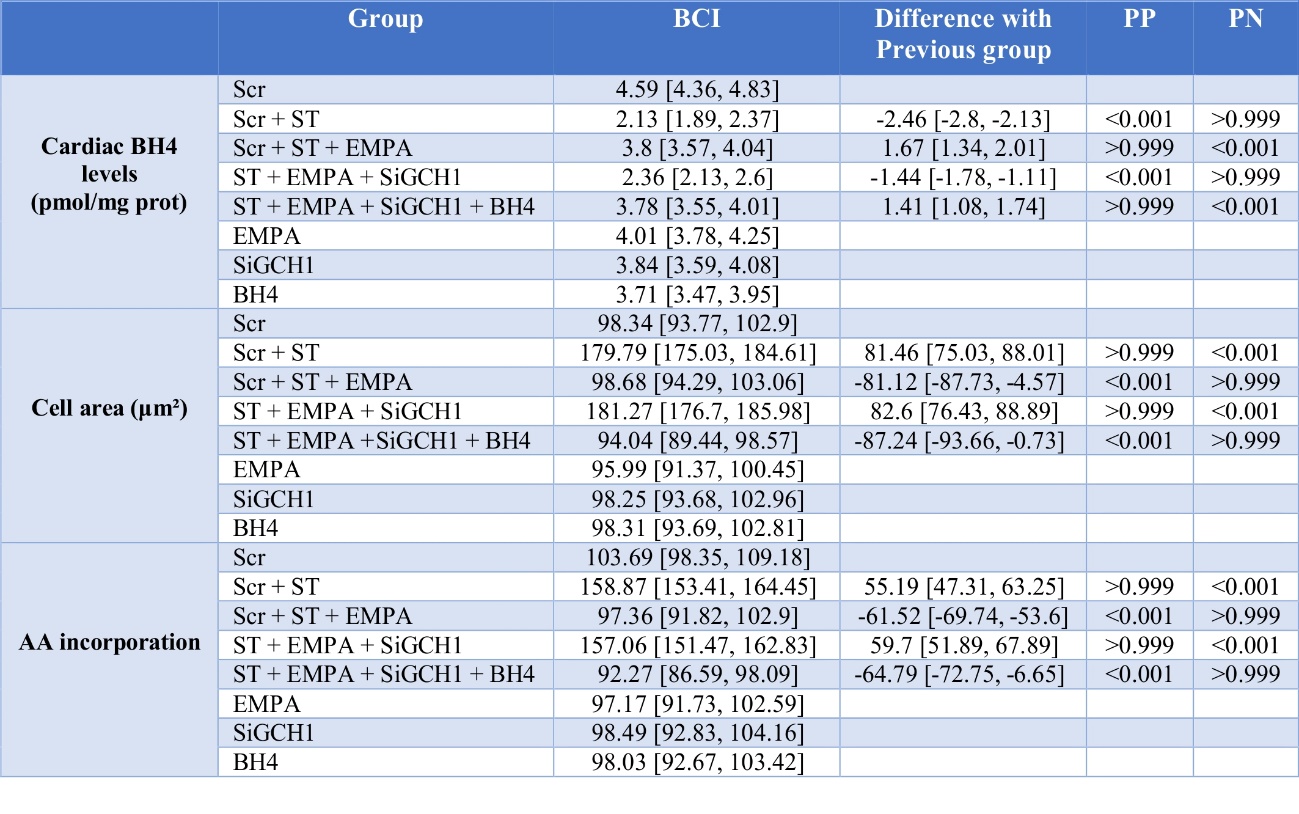
Online table S11. Results respect to figure 6 (Main Manuscript).

AA, aminoacid; BH4, tetrahydrobiopterin; Scr, scramble, ST; stretching;. Data have been represented as described above, in table S1.


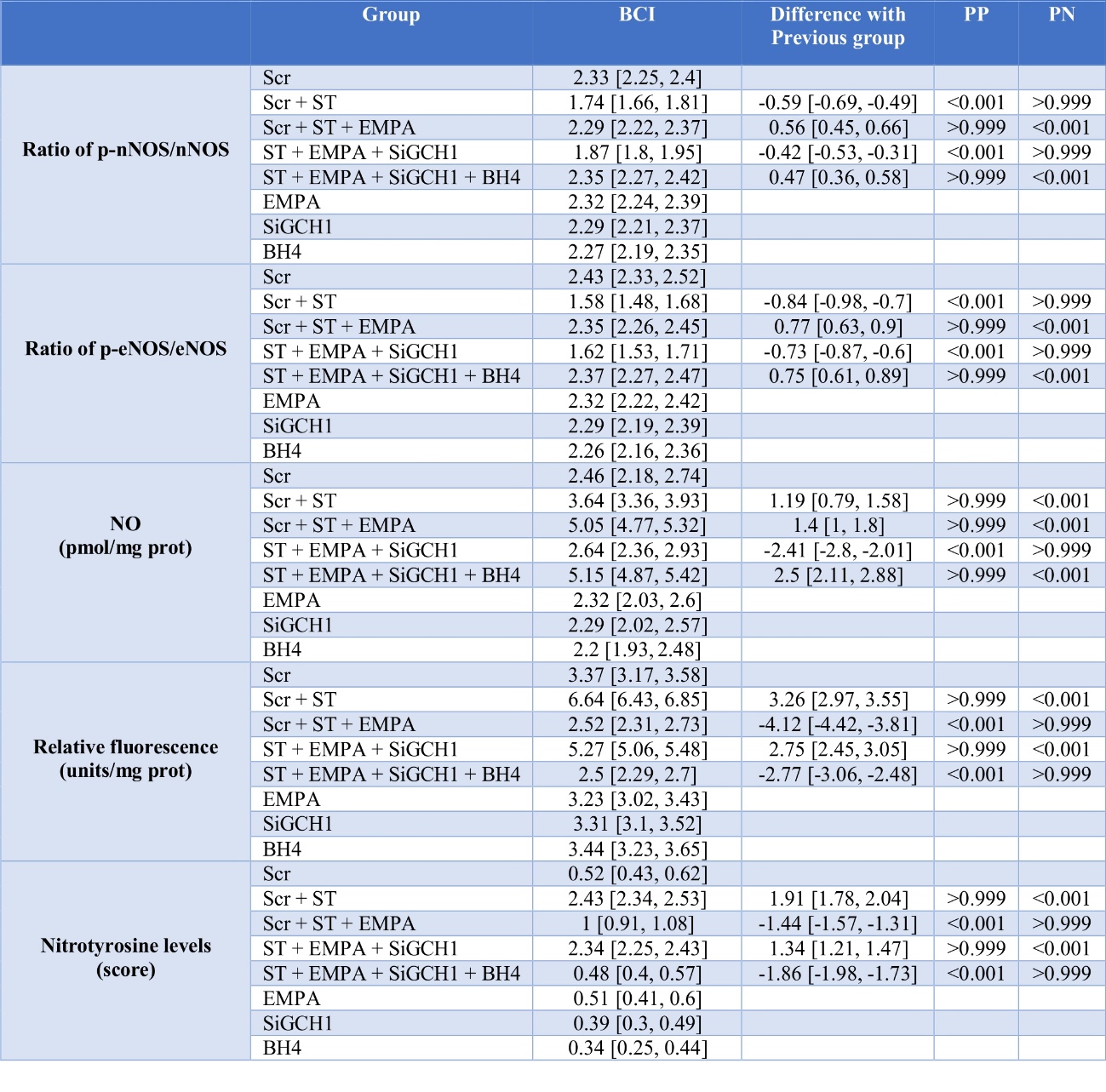
Online table S12. Results respect to figure 7 (Main Manuscript).

BH4, tetrahydrobiopterin; NO, nitric oxide; Scr, scramble, ST; stretching Data have been represented as described above, in table S1.


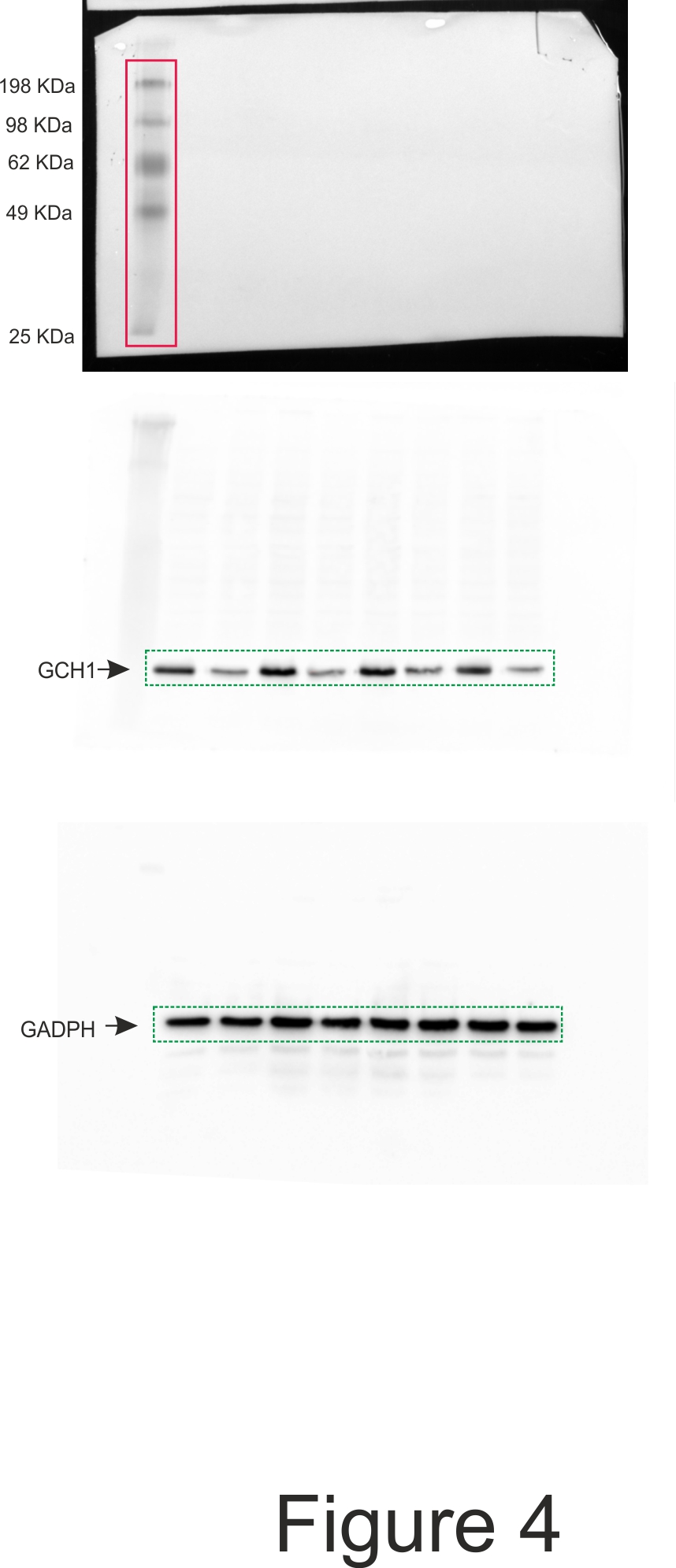
**Representative full Western Blots**

**Full western blot image figure 4b.** Representative pictures of western blots (figure 4, panel b). (Above picture), Molecular weight markers (ThermoFisher, LC5925); (Middle image), GCH1 levels; (Down picture), GADPH levels. Specific bands are selected in green.

**
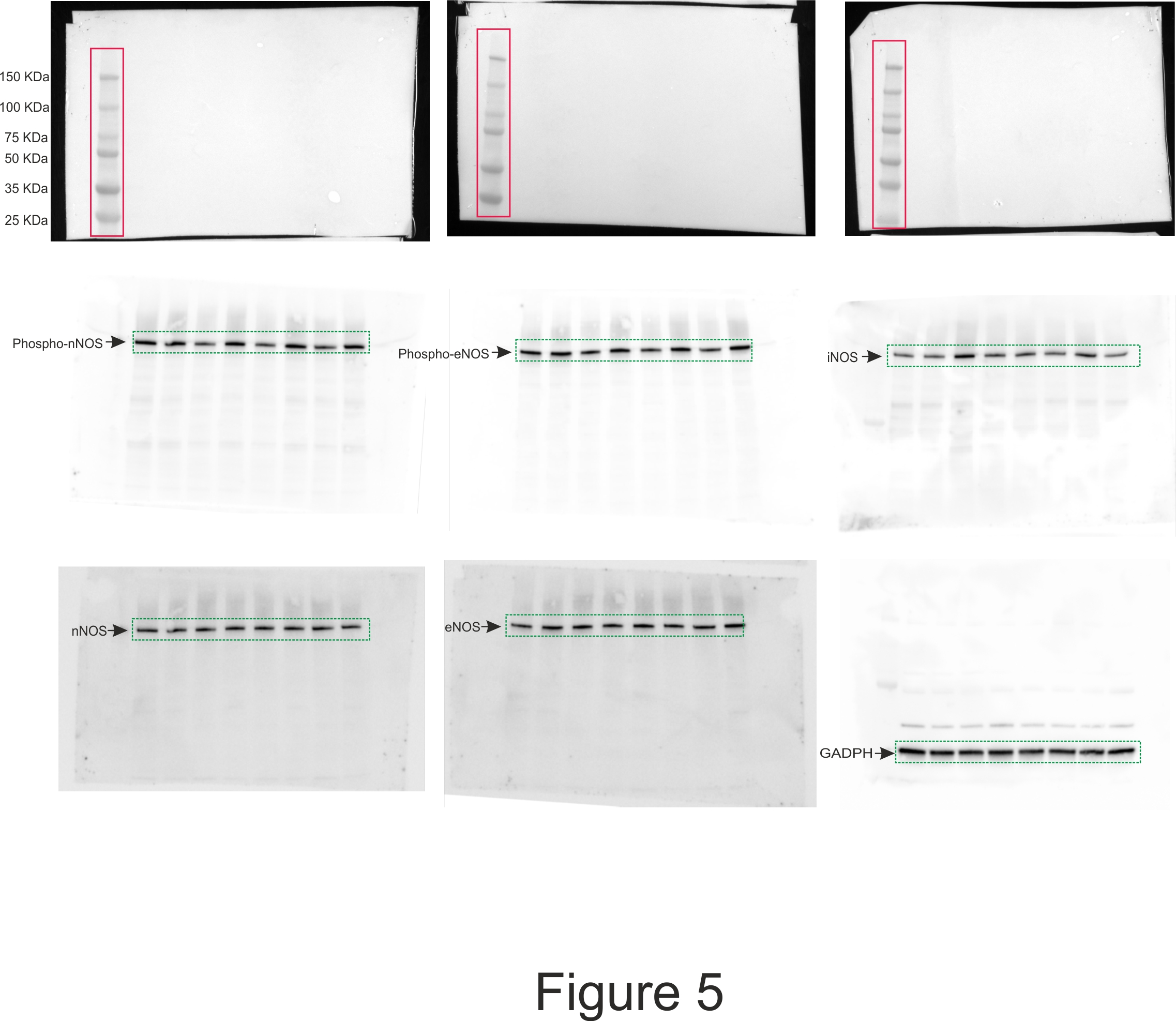
**

**Full western blots image figure 5a-c.** Representative pictures of western blots (figure 5, panels a-c). (Left, figure 5a), (above picture)., Molecular weight markers (Fisher scientific, 10420315); (Middle image), Phospho-nNOS levels; (Down picture), nNOS levels. (Centre, figure 5b), (above picture)., Molecular weight markers (Fisher scientific, 10420315); (Middle image), Phospho-eNOS levels; (Down picture), eNOS levels. (Right, figure 5c), (above picture)., Molecular weight markers (Fisher scientific, 10420315); (Middle image), iNOS levels; (Down picture), GADPH levels. Specific bands are selected in green.

**
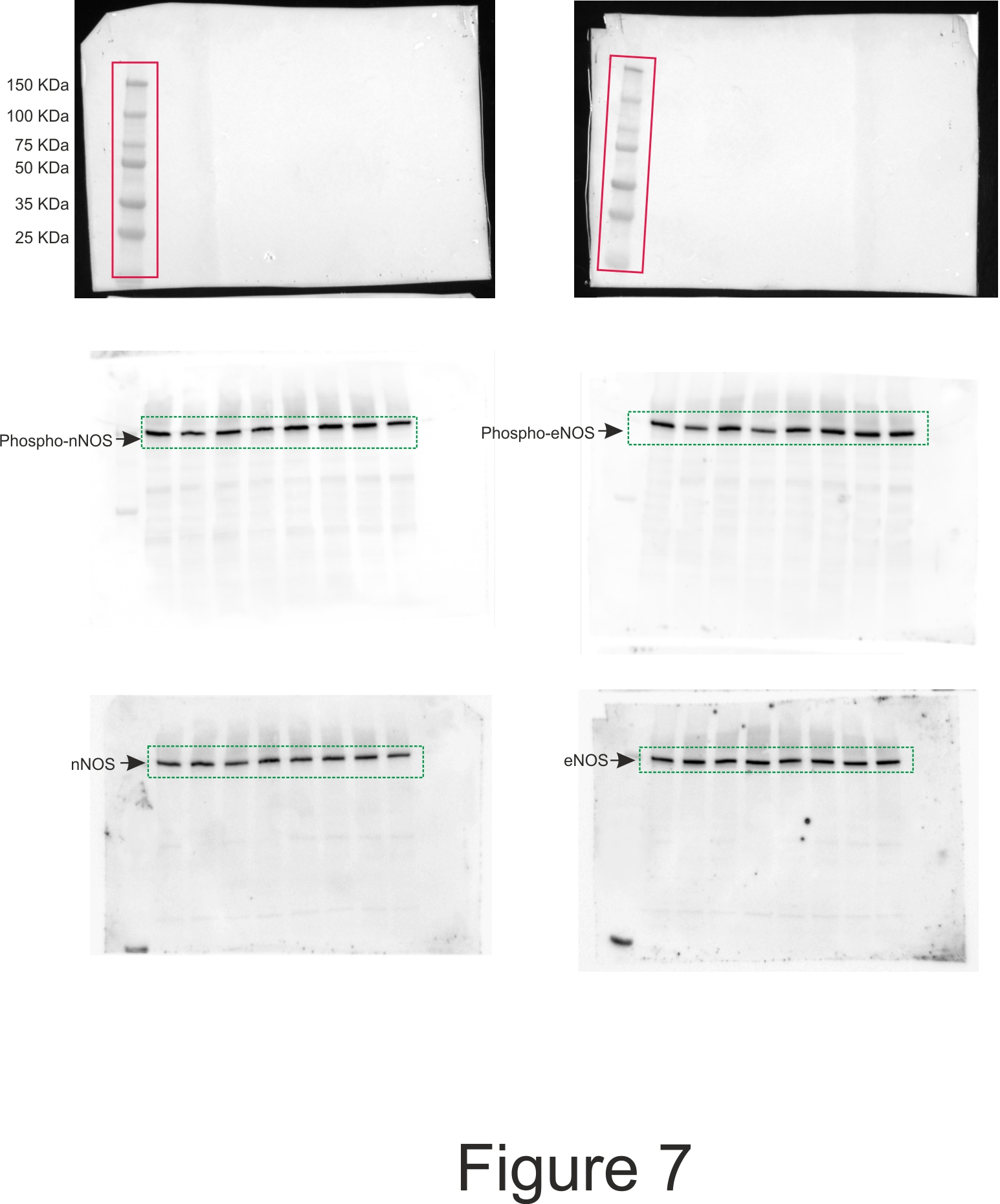
**

**Full western blots image figure 7a-b.** Representative pictures of western blots (figure 7, panels a-b). (Left, figure 5a), (above picture)., Molecular weight markers (Fisher scientific, 10420315); (Middle image), Phospho-nNOS levels; (Down picture), nNOS levels. (Right, figure 5b), (above picture)., Molecular weight markers (Fisher scientific, 10420315); (Middle image), Phospho-eNOS levels; (Down picture), eNOS levels. Specific bands are selected in green.

**References**

1. Thomas, W. P. *et al.* Recommendations for standards in transthoracic two-dimensional echocardiography in the dog and cat. Echocardiography Committee of the Specialty of Cardiology, American College of Veterinary Internal Medicine. *J. Vet. Intern. Med.* **7**, 247–52

2. Sahn, D. J., DeMaria, A., Kisslo, J. & Weyman, A. Recommendations regarding quantitation in M-mode echocardiography: results of a survey of echocardiographic measurements. *Circulation* **58**, 1072–83 (1978).

3. Asensio-Lopez, M. C. *et al.* Yin-Yang 1 transcription factor modulates ST2 expression during adverse cardiac remodeling post-myocardial infarction. *J. Mol. Cell. Cardiol.* **130**, 216–233 (2019).

4. Ng, K. M. *et al.* Empagliflozin Ammeliorates High Glucose Induced-Cardiac Dysfuntion in Human iPSC-Derived Cardiomyocytes. *Sci. Rep.* **8**, 1–13 (2018).

5. Ho, F. M., Liu, S. H., Liau, C. S., Huang, P. J. & Lin-Shiau, S. Y. High glucose-induced apoptosis in human endothelial cells is mediated by sequential activations of c-JUN NH2-terminal kinase and caspase-3. *Circulation* **101**, 2618–2624 (2000).

6. Baumgartner-Parzer, S. M. *et al.* High-glucose-triggered apoptosis in cultured endothelial cells. *Diabetes* **44**, 1323–1327 (1995).

7. Asensio-Lopez, M. D. C. *et al.* Pharmacological inhibition of the mitochondrial NADPH oxidase 4/PKCα/Gal-3 pathway reduces left ventricular fibrosis following myocardial infarction. *Transl. Res.* (2018). doi:10.1016/j.trsl.2018.04.004

8. Andreadou, I. *et al.* Empagliflozin limits myocardial infarction *in vivo* and cell death *in vitro*: Role of STAT3, mitochondria, and redox aspects. *Front. Physiol.* **8**, (2017).

9. Panchapakesan, U. *et al.* Effects of SGLT2 Inhibition in Human Kidney Proximal Tubular Cells—Renoprotection in Diabetic Nephropathy? *PLoS One* **8**, e54442 (2013).

10. Banerjee, I. *et al.* Cyclic stretch of embryonic cardiomyocytes increases proliferation, growth, and expression while repressing Tgf-β signaling. *J. Mol. Cell. Cardiol.* **79**, 133–144 (2015).

11. Bailey, J. *et al.* A novel role for endothelial tetrahydrobiopterin in mitochondrial redox balance. *Free Radic. Biol. Med.* **104**, 214–225 (2017).

12. Oguri, Y. *et al.* Tetrahydrobiopterin activates brown adipose tissue and regulates systemic energy metabolism. *JCI insight* **2**, (2017).

13. Smith, P. K. *et al.* Measurement of protein using bicinchoninic acid. *Anal. Biochem.* **150**, 76–85 (1985).

14. Nar, H. *et al.* Active site topology and reaction mechanism of GTP cyclohydrolase I. *Proc. Natl. Acad. Sci. U. S. A.* **92**, 12120–5 (1995).
